# A pharmacological probe identifies cystathionine β-synthase as a new negative regulator for ferroptosis

**DOI:** 10.1038/s41419-018-1063-2

**Published:** 2018-09-26

**Authors:** Li Wang, Hao Cai, Youtian Hu, Fan Liu, Shengshuo Huang, Yueyang Zhou, Jing Yu, Jinyi Xu, Fang Wu

**Affiliations:** 1 Key Laboratory of Systems Biomedicine (Ministry of Education), Shanghai Center for Systems Biomedicine, Shanghai Jiao Tong University, Shanghai, China; 2 State Key Laboratory of Natural Medicines and Department of Medicinal Chemistry, China Pharmaceutical University, Nanjing, China; 3 State Key Laboratory of Microbial Metabolism, Sheng Yushou Center of Cell Biology and Immunology, School of Life Sciences & Biotechnology, Shanghai Jiao Tong University, Shanghai, China

## Abstract

Cystathionine β-synthase (CBS) is responsible for the first enzymatic reaction in the transsulfuration pathway of sulfur amino acids. The molecular function and mechanism of CBS as well as that of transsulfuration pathway remain ill-defined in cell proliferation and death. In the present study, we designed, synthesized and obtained a bioactive inhibitor CH004 for human CBS, which functions in vitro and in vivo. CH004 inhibits CBS activity, elevated the cellular homocysteine and suppressed the production of hydrogen sulfide in a dose-dependent manner in cells or in vivo. Chemical or genetic inhibition of CBS demonstrates that endogenous CBS is closely coupled with cell proliferation and cell cycle. Moreover, CH004 substantially retarded in vivo tumor growth in a xenograft mice model of liver cancer. Importantly, inhibition of CBS triggers ferroptosis in hepatocellular carcinoma. Overall, the study provides several clues for studying the interplays amongst transsulfuration pathway, ferroptosis and liver cancer.

## Introduction

**Fig. 1:**
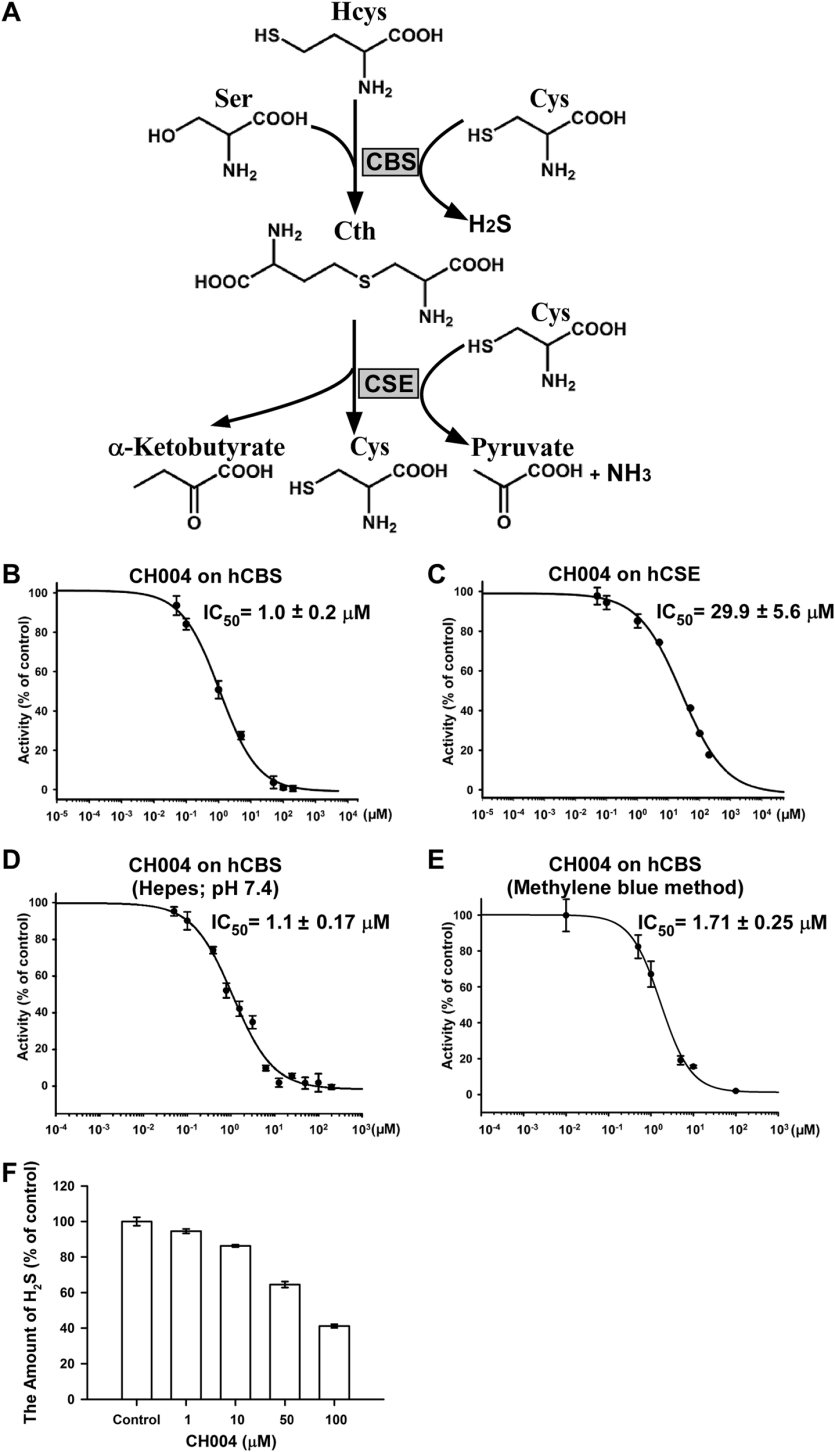
CH004 inhibits the activity of hCBS in the in vitro purified enzyme assays under different assay conditions. **a** A scheme for the cascade enzymatic reactions catalyzed by CBS or CSE. Cth, cystathionine. **b**, **c** Inhibitory effects of CH004 on the activity of hCBS-413 (**b**) or hCSE (**c**). The enzyme activities were monitored for various concentrations of CH004 under the standard conditions (50 mM Tris-HCl, pH 8.6; “Materials and Methods” section). The result is shown as percentages of the control (DMSO, 100%). The data are shown as means ± SDs (*n* = 3). **d** The dose-dependent inhibition of CH004 on the hCBS-413 activity was measured by the tandem-well-based assay under the standard conditions except using 50 mM Hepes buffer (pH 7.4) instead of 50 mM Tris-HCl (pH 8.6). Means ± SDs (*n* = 3). **e** The dose-dependent inhibition of CH004 on the CBS activity measured by methylene blue method. The CBS enzyme assay was performed with a 50 μl assay buffer in an Eppendorf tube under the standard assay conditions for the 192-tandem-well based assay, i.e., 50 μl 50 mM Tris-HCl buffer (pH 8.6) containing 150 nM hCBS-FL, 200 μM S-adenosyl-L-methionine, 4 mM L-Cys and 4 mM d,l-Hcys, in the presence of 0 (DMSO control), 0.01, 0.5, 1, 5, 10 or 100 μM CH004. After 80 min, the assay was stopped by added 10 μL 10% ZnAC and the generated H_2_S was quantified by *N*,*N*-dimethyl-p-phenylenediamine to give an absorbance at 670 nm (for details, see Materials and Methods section; ref. ^53^). The data are presented as percentages of control (DMSO, 100%) and shown as means ± SDs (*n* = 3. The dose-dependent curves were fitted to the data points with GraphPad Prism 5. **f** The interference of CH004 with H_2_S at micomolar concentrations. CH004 at the indicated concentrations was mixed with 100 μM NaHS in the absence of CBS under the assay conditions (“Materials and Methods” section). The released H_2_S was quantified with DTNB by using the 192-tandem plate. The data are presented as percentages of control (DMSO, 100%) and shown as means ± SDs (*n* = 3)

The transsulfuration pathway for sulfur amino acids in the human body relies mainly on enzymatic reactions of cystathionine β-synthase (CBS) and cystathionine γ-lyase (CSE)^1,2^, two vitamin B_6_-dependent enzymes. CBS could metabolize l-homocysteine (Hcys) into cystathionine (CTH), which is the main source for CSE to generate intracellular l-cysteine (Cys; Fig. 1a)^2^. Recently, mercaptopyruvate sulfurtransferase (MST) has also been reported to degrade Cys to produce pyruvate^3^. All three enzymes could employ Hcys, Cys, CTH, mercaptopyruvate or their combinations as a substrate to produce hydrogen sulfide (H_2_S)^4^. CBS and CSE are commonly known to have specific tissue distributions, i.e., CBS mainly produces H_2_S in the brain, whereas CSE mainly produces H_2_S in the cardiovascular system^5–7^. These two well-known sulfide-producing enzymes are also concomitantly present in many tissues, e.g., the liver and kidney^8–11^. Moreover, CBS has been reported to be predominantly expressed in HepG2 and A549 cell lines, though substantial CSE is also present^12,13^.

Cystine starvation has been reported to induce the cell death in various types of cancer cells^14^. The mechanism behind this phenomena is thought to be that the decreased uptake of extracellular cystine by *X*_c_^-^ cystine/glutamate antiporter could suppress the intracellular level of glutathione (GSH) and increase the amount of reactive oxygen species (ROS), which are closed coupled with the ferroptosis, a recently recognized non-apoptotic cell death process^15,16^. Consistently, the degradation of extracellular cystine with an engineered enzyme of CSE has also been demonstrated to suppress the growth of various types of cancers in cells and in vivo^17,18^.

Besides uptaking cystine from extracellular matrix, mammalian cells could also employ the transsulfuration pathway to synthesize cysteine *de novo* for the generation of GSH^19^. Recently, genetic knock-down of cysteinyl-tRNA synthetase (CARS) was found to blunt the ferroptotic cell death triggered by erastin that is an inhibitor of system *X*_c_^-^, via an upregulation of the expression of CBS in fibrosarcoma HT1080 cells, and the knock-down of CBS could re-sensitize the CARS-knock-down cells to erastin^20^. However, whether the activity of CBS alone is sufficient to regulate ferroptosis remains largely unknown.

Abnormal de-regulation of CBS in Down syndrome, colon cancer, ovarian cancer or glioma as well as breast and lung cancer has been reported^21–25^. Considering cystine and the transsulfuration pathway is implicit in cell deaths^14,17,20^, it seems to be rational to develop pharmacological inhibitors of CBS to explore its function and mechanism in cells and develop new treatments for related cancers^4^.

The widely-used inhibitors of CBS are hydroxylamine (HA) and aminooxyacetic acid (AOAA)^26–29^. These inhibitors are non-selective and low-affinity inhibitors for CBS, since they also inhibit other vitamin B_6_-dependent enzymes^29^. Recentlly, great efforts have been made to identify new inhibitors of CBS^30–35^, however, few validated bioactive and selective inhibitors of CBS in cells have been reported to date. The application of CBS inhibitors to probe the underlying function and mechanism of CBS or transsulfuration pathway and to deliver new therapeutic treatment for related diseases is thus in the infant stage.

In this study, we report a potent and bioactive inhibitor of CBS; this new inhibitor has an IC_50_ of 1 μM and preferably inhibits the activity of CBS rather than CSE in vitro and in vivo. Utilizing this pharmacological probe, we demonstrated that the selective inhibition of CBS could suppress cell proliferation, cause cell cycle arrest at S phase and significantly reduce in vivo tumor growth in tumor xenograft mice model. Importantly, we were able to demonstrate that the underlying mechanism of cell death triggered by CBS inhibition in HepG2 cells is via ferroptosis, suggesting that CBS has a previously unreported function in ferroptosis.

## Results

### Identification of potent inhibitors for CBS

To identify new inhibitors with better potency and selectivity for human CBS (hCBS), we designed and synthesized a few derivatives of polycyclic-ketone-based inhibitors of CBS, which we have previously uncovered through a high-throughput screening assay with a 192-tandem-well plate^30^. The compound CH004 (Supplementary Table 1) comes out to be the most potent and selective inhibitor of hCBS, which has an IC_50_ of ~1 μM and ~0.59 μM for hCBS-413 (a C-terminal truncated hCBS 1–413) and hCBS full-length (CBS-FL), respectively, and holds a selectivity of ~ 30-fold with respect to hCSE or ~ 400-fold with respect to human dopa decarboxylase (hDDC; Fig. 1b, c and Supplementary Table 1), another vitamin B_6_-dependent enzyme^36^. CH004 also displays a similar IC_50_ for CBS if the assay is carried out under a different assay buffer and pH condition (pH 7.4, Fig. 1d) or is measured by a different H_2_S detecion method using methylene blue (Fig. 1e).

Since H_2_S may react with compounds in its nucleophilic anionic form^12,33^, we performed an interfering assay with NaSH, a commonly-used H_2_S donor to exam this possibility (Fig. 1f). The results showed that CH004 did not absorb much the released H_2_S from NaSH at 1 or 10 μM, however, we did observe that it trapped a significant amount of H_2_S at a concentration starting from 50 μM. The latter suggests that CH004 may behave via multiple mechanisms to interfere with the H_2_S or CBS.

To determine the mode of action of CH004, we performed rapid dilution, enzyme kinetics and surface plasmon resonance (SPR) experiment as well as mutagenesis (Supplementary Figure 2 and Table 2). The result indicates that CH004 binds reversibly to hCBS, as examined by diluting the enzyme-plus-inhibitor solutions with the assay buffer (Supplementary Figure 2a). In the kinetic studies, this potent inhibitor showed noncompetitive inhibition toward PLP (with the αK_i_ ~15.2 μM) and mixed-type inhibition toward both Cys and Hcys (with the αK_i_ ~26.4 μM for Cys or αK_i_’ ~7.2 μM for Hcys) (Supplementary Figure 2b–d). Importantly, we confirmed by surface plasmon resonance (SPR) measurements that CH004 physically binds to CBS with a K_D_ value of 0.6 ± 0.3 μM, which is similar to its IC_50_ (~1 μM; Supplementary Figure 2e). Furthermore, to verify the binding site and specificity of CH004, we produced and characterized different mutants of hCBS, i.e., T146A, S147A, Q222A and Y223F, which were proposed to interact with CBS inhibitors or serine (Ser) substrate^30,37^. The inhibitory effects of CH004 were largely abolished when tested against the Q222A mutant (23-fold increases in IC_50_ value; Supplementary Table 2 and Supplementary Figure 3a), indicating that Gln222 is largely in charge for the binding of CH004.

### CH004 selectively inhibits the activity of CBS, but not CSE, in cells at low μM concentrations

**Fig. 2:**
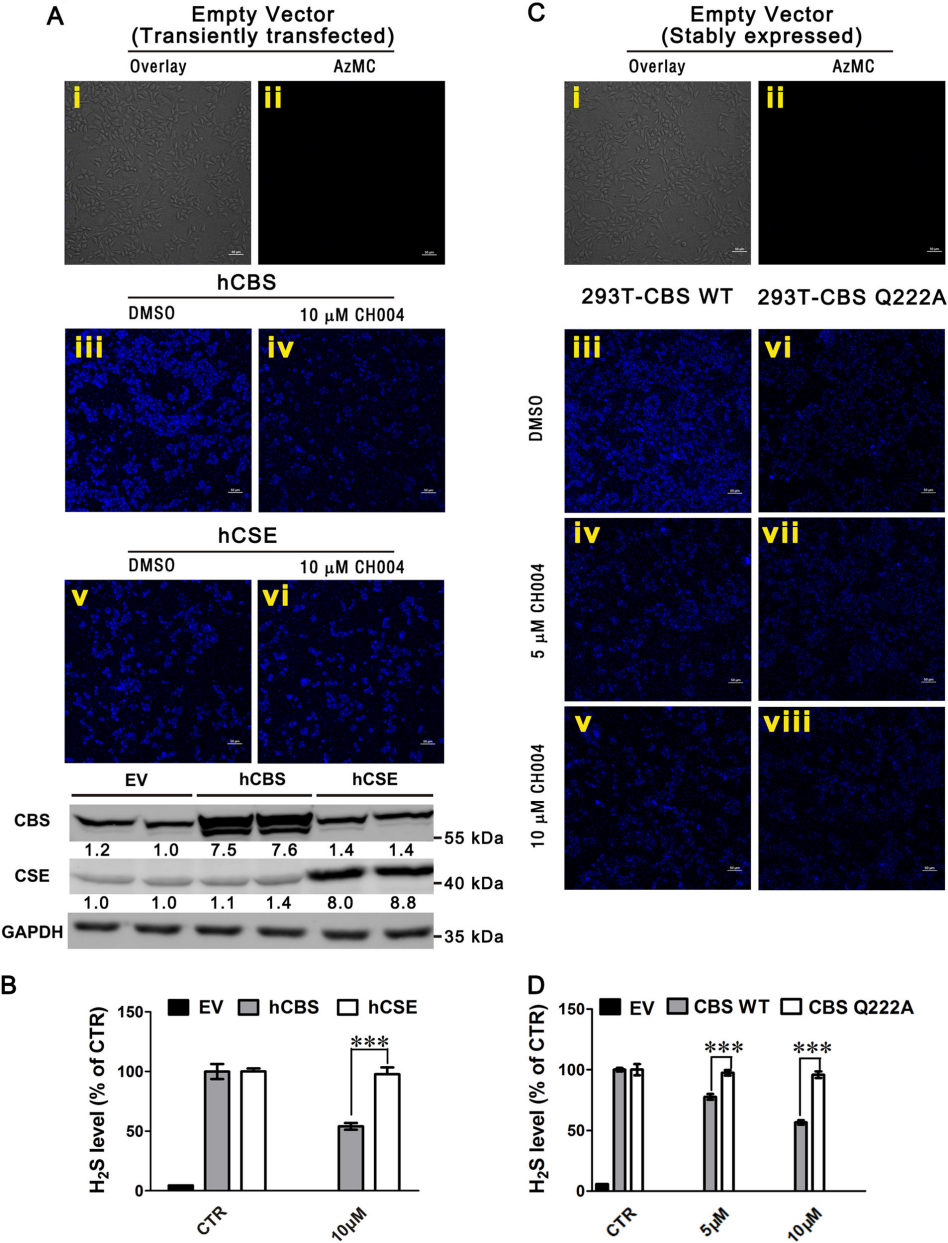
CH004 selectively inhibits hCBS, but not hCSE, in cells. **a**, **b** CH004 significantly reduces the activity of hCBS but not of hCSE in HEK293T cells. HEK293T cells were transiently transfected with empty vector (EV, i and ii), hCBS-FL (iii and iv) or hCSE (v and vi) plasmids, followed by incubation with DMSO (i–iii and v) or 10 μM CH004 (iv and vi) for 8 h before staining with 50 μM AzMC, a H_2_S-specific probe (“Materials and Methods” section). For larger fluorescent and bright-field images, please see Supplementary Figure 4. The expression levels of hCBS or hCSE in HEK293T cells after transient transfections were determined by western blotting using an anti-CBS antibody (3E1, 1:2000; Abnova) or anti-CSE antibody (1:500, GeneTex). The densities of protein bands for hCBS or hCSE in the blots were quantified with the ImageJ software (National Institutes of Health, Bethesda, MD, USA) and expressed as ratios of respective control (EV group). All immunoblots are representative of at least two independent experiments using GAPDH as the loading control. The fluorescent images of the H_2_S (blue) were accordingly quantified (“Materials and Methods” section) with ImageJ. The quantified levels for H_2_S (blue) were showed in (**b**). The DMSO groups of the controls (CTR), which are expressing of hCBS or hCSE, were normalized to 100% and the levels of H_2_S in CH004-treated or EV group are shown as relative percentages. **c**, **d** CH004 dose-dependently suppresses the activity of hCBS WT without affecting the activity of CH004-insensitive mutant. HEK293T cells stably expressing EV (i and ii), hCBS-FL wild type (293T-CBS WT; iii–v) or CBS-FL mutant (293T-CBS Q222A, vi–viii) were treated with DMSO (i–iii and vi), 5 μM CH004 (iv, vii) or 10 μM CH004 (v, viii) for 8 h before staining with the AzMC. For larger fluorescent and bright-field images, please see Supplementary Figures 5–6. The quantified levels for H_2_S (blue) were showed in (**d**). The DMSO groups of the controls (CTR), which are expressing of hCBS-FL wt or Q222A mutant, were normalized to 100% and the levels of H_2_S in CH004-treated or EV group are shown as relative percentages. A representative fluorescence image from three independent experiments is shown. Means ± SDs (*n* = 3). Statistical analyses were performed on the raw data for each group by one-way (**b**) or two-way (**d**) ANOVA with Bonferroni post-tests. ***p < 0.001. Bars, 50 μm

CH004 was found to greatly reduced the amount of H_2_S at 10 μM in HEK293T cells expressing hCBS (~ 50% decrease; Fig. 2a, b). By contrast, CH004 did not interfere with the production of H_2_S in cells expressing hCSE (Fig. 2a, b and Supplementary Figure 4). Since the expression levels of hCBS and hCSE was found to be similar in the transfected cells (Fig. 2a), it suggests that the inhibition of CH004 on CBS is specific in cells. To further demonstrate the on-target effect of CH004 in cells, HEK293T cells stably expressing hCBS WT or Q222A mutant (insensitive to CH004; Supplementary Table 2 and Supplementary Figure 2) were incubated with CH004. CH004 caused a 22 and 43% decrease in H_2_S in 293T-CBS WT cells at 5 and 10 μM, respectively, whereas no significant decrease in the cells expressing the Q222A mutant (Fig. 2c–d and Supplementary Figure 5–6). In a sharp contrast, the H_2_S of HEK293T cells expressing CBS Q222A, was substantially decreased by HA (Supplementary Figure 7a-b), the known unspecific inhibitor of CBS^29^. Consistently, the activity of purified CBS Q222A could be suppressed by HA (Supplementary Figure 7c). These results indicated that CH004 selectively inhibits hCBS and reduces H_2_S in cells.

**Fig. 3:**
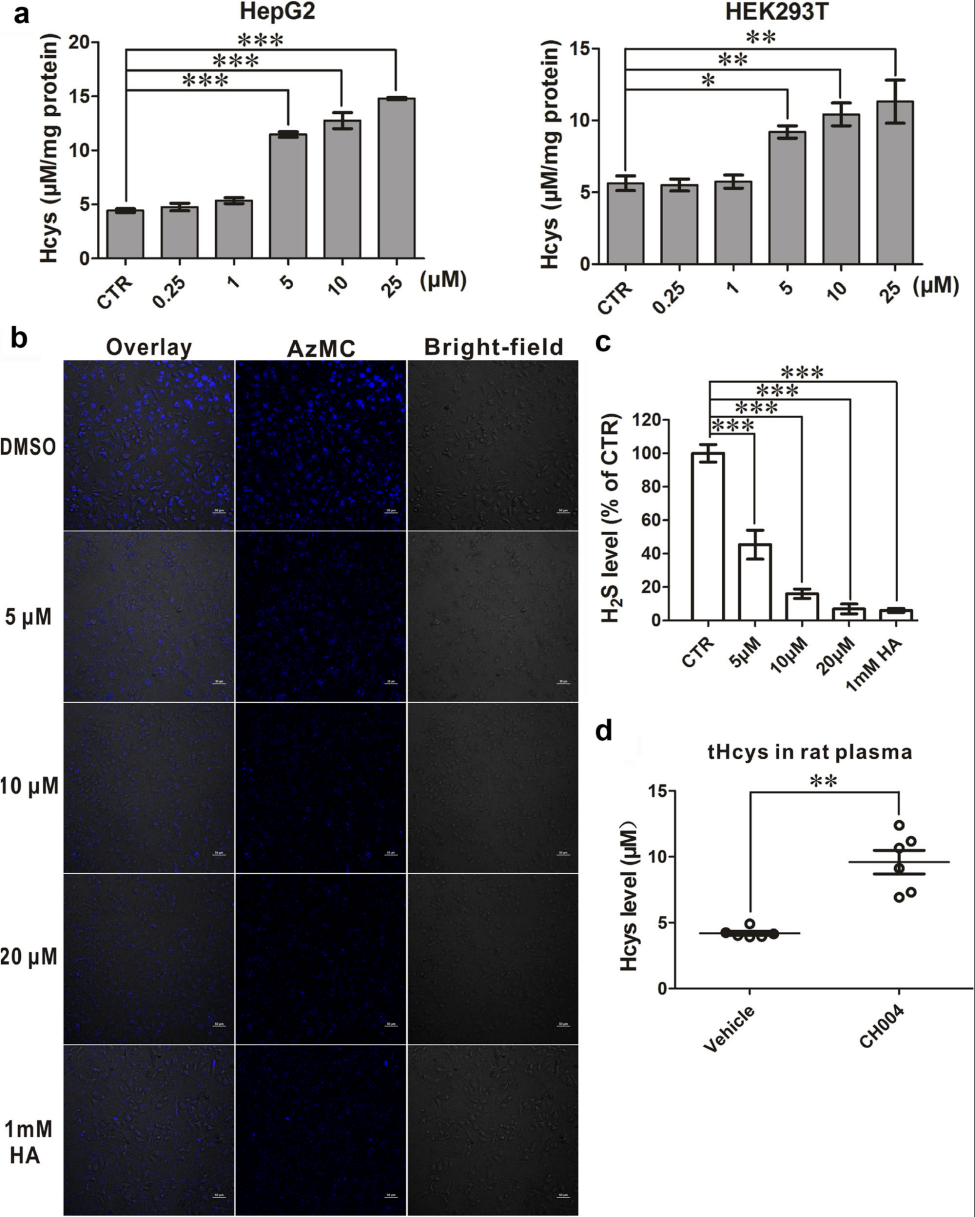
The effect of CH004 on the activity of endogenous hCBS in cells. **a** CH004 dose-dependently elevates the levels of Hcys in HepG2 and HEK293T cells. HepG2 cells or HEK293T cells were incubated with indicated CH004 or DMSO (control) for 12 h. The amount of total Hcys in cells was measured by ELISA using Axis^®^ Homocysteine EIA kit (IBL, AX51301) (“Materials and Methods” section). The concentrations of Hcys are expressed as μM per mg protein and presented as means ± SDs (*n* = 4). **b**, **c** CH004 dose-dependently inhibits the production of H_2_S generated by hCBS in HepG2. HepG2 cells were treated with indicated DMSO, CH004 or HA for 8 h before the staining with AzMC probe. The amount of the H_2_S (blue) was quantified and is shown in (**c**). Bars, 50 μm. Control (CTR), DMSO group (100%). A representative fluorescence image from three independent experiments is shown. Means ± SDs (*n* = 3). **d** CH004 elevates Hcys in the peripheral blood of rats. The total Hcys of the blood samples collected from the anesthetized rats at the 75 min time point (Materials and Methods) were quantified by ELISA (see above). Means ± SDs (*n* = 6). Statistical analyses were performed on the raw data for each group by one-way ANOVA with Bonferroni post-tests. *p < 0.05; **p < 0.01; ***p < 0.001

We also tested the effects of CH004 on the activity of endogenous CBS by incubating with HepG2 (a human liver carcinoma cell line) or HEK293T cells, which have been reported to express high levels of CBS^38,39^. CH004 dose-dependently elevated the concentrations of Hcys, the substrate for hCBS^40^, and 5 or 10 μM CH004 was able to increase Hcys by ~ 2-fold in HepG2 and HEK293T cells (Fig. 3a), strongly demonstrating that it blocks the transsulfuration pathway of Hcys. In line with the increase in cellular Hcys, H_2_S in HepG2 cells was dose-dependently blocked by CH004 (Fig. 3b). Remarkably, treatment with 5 μM CH004 already showed a ~50% decrease in H_2_S, and at 20 μM CH004 achieved a ~90% decrease. Conversely, AOAA cannot suppress the production of H_2_S in HEK293T cells stably expressing hCBS or in HepG2 cells at 1 mM, and only decreases cellular H_2_S by up to ~ 30% at 10 mM (Supplementary Figures 8–9). The potency of CH004 on inhibiting the production of H_2_S in cells exceeds at least 1000-fold when compared with AOAA, suggesting it is a highly potent inhibitor for CBS in cells.

Importantly, CH004 at a dose of 3 mg/kg could increase ~ 2-fold the level of Hcys in a rat hemorrhagic shock model, while it does not affect the mean arterial pressure (MAP), an indicator for in vivo CSE inhibition (Fig. 3d and Supplementary Figure 10; ref. ^41^). It demonstrates that CH004 is an effective inhibitor in vivo, selectively inhibits CBS and hardly affects CSE in rats.

### Inhibition of CBS activity suppresses cell proliferation and causes cell cycle arrest

**Fig. 4:**
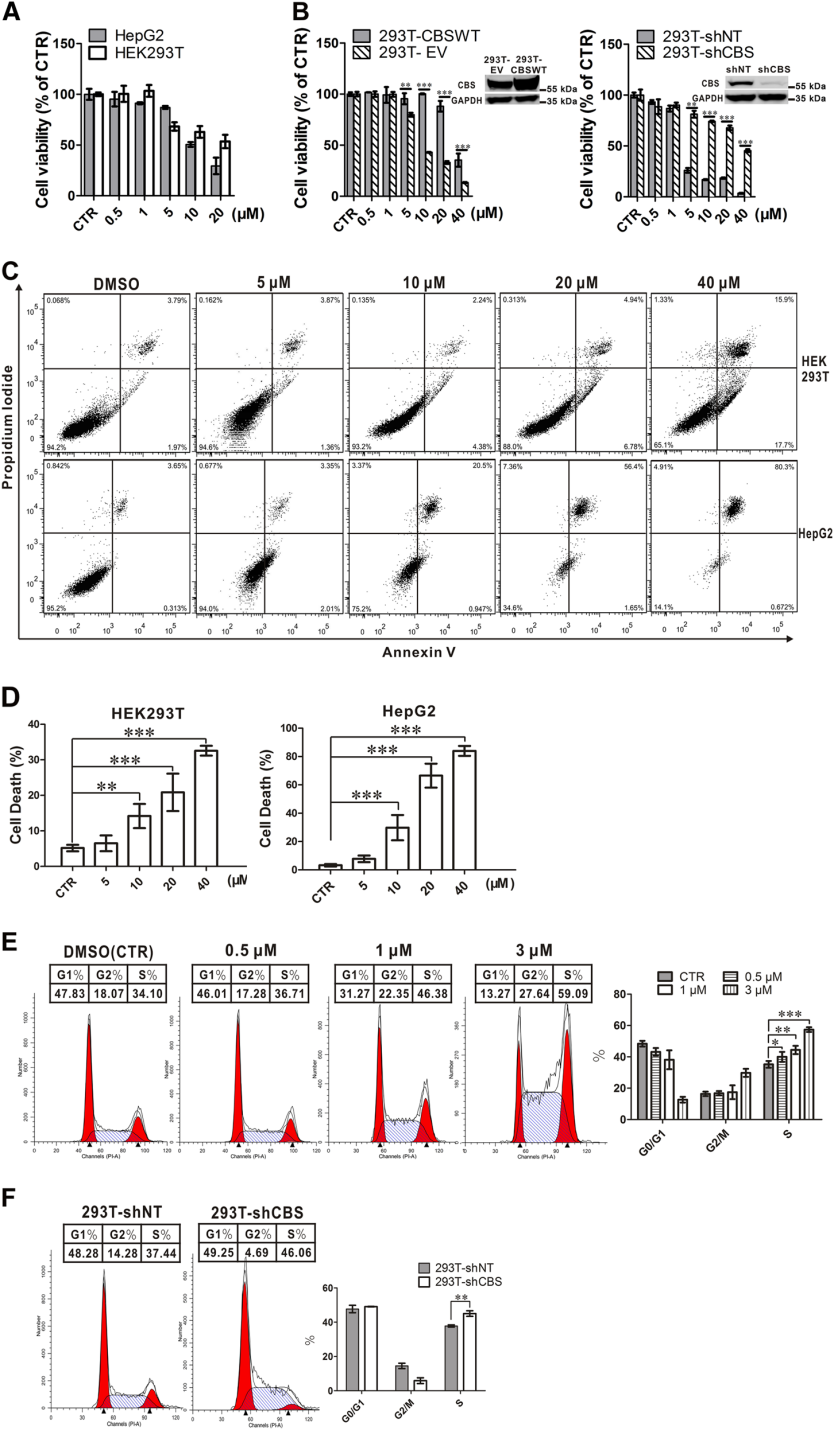
CH004 arrests the cell cycle at the S phase. **a** CH004 reduces the cell viability of HEK293T and HepG2 cells. HEK293T or HepG2 cells were treated with indicated concentrations of CH004 for 24 h before the cell number was analyzed using the CellTiter96® Aqueous One Solution Cell Proliferation Assay (Promega, Materials and Methods). The means at each concentration of one representative experiment are shown as percentages of DMSO (control, 100%). Means ± SDs (*n* = 3). **b** Overexpression of hCBS or knock-down of hCBS counteracts the growth inhibition exerted by CH004 in HEK293T cells. Indicated HEK293T stable cell lines were treated with the CH004 or DMSO for 24 h. Then, the cell viability was quantified with the CellTiter 96^®^ Aqueous One Solution Cell Proliferation Assay under the standard protocol (Promega). The means at each concentration are shown as percentages of their respective DMSO controls (100%). Means ± SDs (*n* = 3). The efficacy for CBS overexpression and knock-down in HEK293T cells was determined by Western blotting using an anti-CBS antibody (3E1, 1:2000; Abnova). **c** CH004 induces cell death in HEK293T and HepG2 cells. HEK293T or HepG2 cells were incubated with the indicated concentrations of CH004 or DMSO for 12 h before analysis using a FITC Annexin V Apoptosis Detection Kit (BD bioscience, 556547) on an LSR Fortessa flow cytometer. The means of percentages of dead cells (DMSO, 100%) from three independent samples are shown in **d**. Means ± SDs (*n* = 3). **e**, **f** Pharmacological inhibition or genetic knock-down of hCBS in HEK293T cells arrests the cell cycle at S phase. HEK293T cells were incubated with indicated compounds for 12 h, and the DNA content was quantified with propidium iodide to analyze the cell cycle distribution by flow cytometry (**e**). The cell cycle distributions for HEK293T-shNT and HEK293T-shCBS cells were accordingly analyzed and are shown in **f**. One representative diagram is shown for each condition. The mean percentages of various cell phases from three independent samples are shown in the respective right panels. Means ± SDs (*n* = 3). Statistical analyses were performed by two-way ANOVA with Bonferroni post-tests. *p < 0.05; **p < 0.01; ***p < 0.001. All the experiments were independently repeated twice and one representative result is present

With a bioactive and selective inhibitor of CBS in hand, we examined the effect of CH004 on the proliferation of HepG2 and HEK293T cells as well as hepatocellular carcinoma Huh7 and H22, pancreatic cancer Panc-28, colon cancer HCT116 and breast cancer MDA-MB-231 cells. CH004 could suppress the proliferation of all tested cells with an IC_50_ range of 4 to 25 μM. HCT116 was the most affected cell line with an IC_50_ of 4 μM amongst tested cell lines (Fig. 4a and Supplementary Figure 11). For HepG2, Huh7, H22 and HEK293T cells, we observed that CH004 has an IC_50_ value of ~ 11, 12, 13 and 17 μM, respectively. To confirm that CH004-mediated inhibitory effects are due to the specific inhibition of hCBS, we generated hCBS-overexpressed (293T-CBS WT) stable cell lines by transducing HEK293T cells with lentivirus carrying hCBS WT genes. The overexpression of CBS in HEK293T cells counteracted the inhibitory effect of CH004 by 3-fold (Fig. 4b and Supplementary Figure 12a), suggesting that CBS is the cellular target of CH004 in terms of its anti-proliferation effect. In supporting the pros of CBS on the proliferation, knock-down of CBS indeed substantially retards cell proliferation in HEK293T cells (Supplementary Figure 12c, d). Surprisingly, CBS knock-down drastically increased the IC_50_ of CH004 by 15-fold (Fig. 4b and Supplementary Figure 12b), indicating the cell proliferation of CBS-knock-down cells is decoupled with the activity of CBS. Importantly, CH004 could largely retard the colony formation of HepG2 cells with an IC_50_ of ~8 μM (Supplementary Figure 12e–g).

**Fig. 5:**
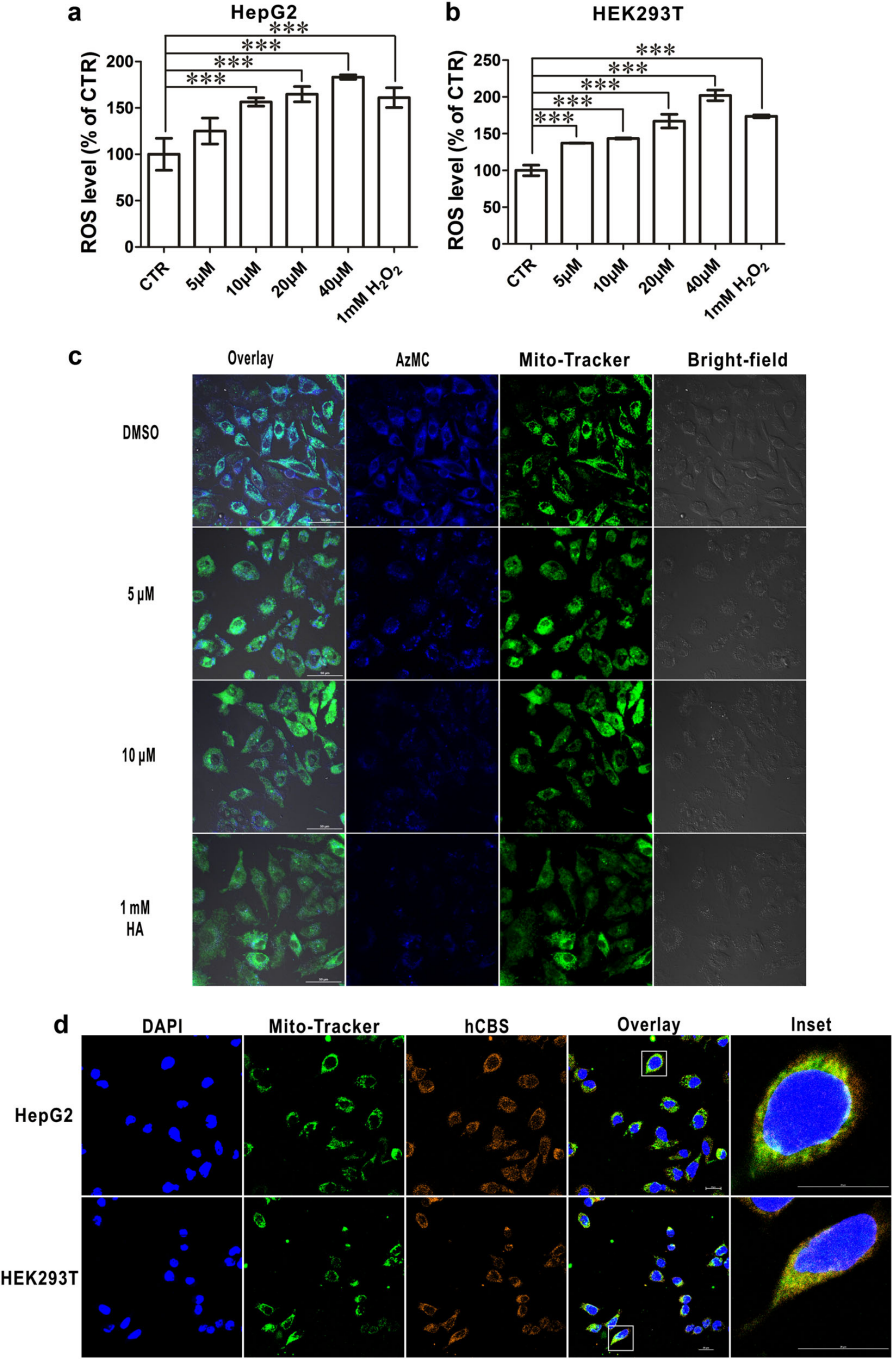
In situ reduction of CBS activity and H_2_S in mitochondria by CH004 is coupled with an increase of intracellular ROS. **a**, **b** CH004 dose-dependently elevates ROS levels in both HepG2 (**a**) and HEK293T cells (**b**). The cells were treated with CH004 for 12 h or with 1 mM H_2_O_2_ for 30 min before the staining with DCFH-DA (Molecular Probes) and the analyzsis by flow cytometry (“Materials and Methods” section). Means ± SDs (*n* = 3). The experiments were independently repeated twice and one representative result is present. Statistical analyses were performed by one-way ANOVA with Bonferroni post-tests. ***p < 0.001. **c** CH004 dose-dependently decreases mitochondrial H_2_S. HepG2 cells were incubated with indicated inhibitors for 8 h before staining with the mitochondria tracker Mito-tracker^®^ Green (Invitrogen) and the AzMC probe. Cyan represents the colocalization of H_2_S (blue) and the mitochondria tracker (green). Bars: 50 μm. **d** hCBS co-localizes with the mitochondria in HepG2 and HEK293T cells. HepG2 and HEK293T cells were stained with an anti-CBS antibody (3E1, 1:200; Abnova) for hCBS (red), Mito-tracker^®^ Green for the mitochondria (green) and DAPI for the nucleus (blue). Yellow, colocalization of CBS (red) and mitochondria tracker (green). Bars: 20 μm. All images are representative of three independent experiments

The advantages of the pharmacological probe enabled us to study the immediate effect of CBS on cell death and the cell cycle in a dose-dependent manner. The number of dead HepG2 or HEK293T cells increased in a dose-dependent manner with CH004 treatment (Fig. 4c). After exposure for 12 h, 40 μM CH004 caused a ~ 30% and ~ 80% increase in the cell death of HEK293T and HepG2 cells (Fig. 4d), respectively. To specify the role of CBS in the cell cycle, we analyzed the cell cycle phases of HEK293T by flow cytometry. CH004 at a concentration of 3 μM arrested the proliferation of HEK293T cells at the S phase, as reflected by a more than 20% increase in cell number in the S phase (Fig. 4e). A similar effect was also observed when CBS was knocked down (Fig. 4f), implying that the activity of CBS is important for the cell cycle. Concomitantly, cellular ROS was dose-dependently elevated with a maximum increase of ~ 2-fold in both HepG2 and HEK293T cells when CBS activity was suppressed by CH004 (Fig. 5a, b). Interestingly, this effect is actually accompanied by a decrease in H_2_S, which seems to be mainly produced in the mitochondria (Fig. 5c). Indeed, we found that substantial CBS appeared in the mitochondria in both HepG2 and HEK293T cells, as indicated by immunofluorescence colocalization studies with two different CBS antibodies (Fig. 5d and Supplementary Figure 13). This observation seems to be consistent with recent findings that CBS is present in mitochondria and has an internal mitochondrial localization sequence^22,42,43^. Taken together, these results indicated that the activity of CBS is involved in cell proliferation and likely plays a critical role in the S phase.

### Inhibition of CBS activity by CH004 triggers ferroptosis in HepG2 cells

**Fig. 6:**
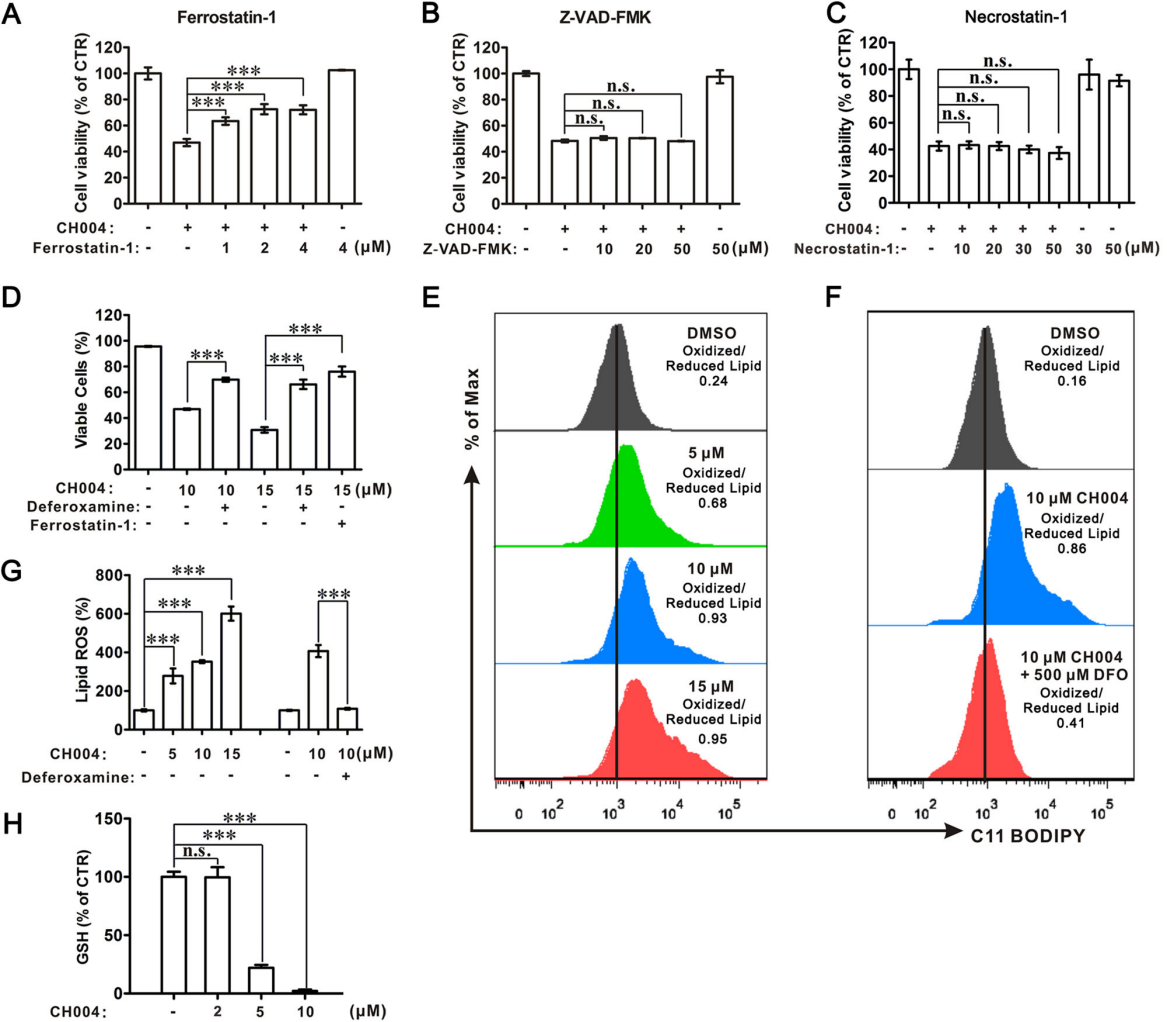
The effects of CH004 on cell viability and lipid ROS could be prevented by ferroptosis inhibitors. **a**–**c** CH004-induced cell death can be prevented by ferroptosis inhibitors but not by apoptosis or necroptosis inhibitors in HepG2 cells. HepG2 cells were treated with 10 μM CH004 in the presence or the absence of the indicated concentrations of the ferroptosis inhibitor ferrostatin-1 (**a**), apoptosis inhibitor Z-VAD-FMK (**b**) or necroptosis inhibitor necrostatin-1 (**c**) for 24 h before analysis of the cell viability (CellTiter96® Aqueous One Solution Cell Proliferation Assay, Promega). The means at each condition are shown as percentages of DMSO (control, 100%). Means ± SDs (*n* = 3). **d** Analysis of viable cells treated with CH004 in the presence or the absence of ferroptosis inhibitor by flow cytometry. HepG2 cells were treated with CH004 in the presence or the absence of 500 μM deferoxamine or 2 μM ferrostatin-1 for 24 h, before analysis using a FITC Annexin V Apoptosis Detection Kit on an LSR Fortessa flow cytometer. Typical bright-field images for cells were recorded and are shown in Supplementary Figure 14a, and the flow cytometry charts are presented in Supplementary Figure 14b. The percentages of viable cells were defined based on the Annexin V-PI double-negative-stained cells in three independent samples. Means ± SDs (*n* = 3). **e**–**g** CH004 increases lipid ROS in a dose-dependent manner, which can be counteracted by deferoxamine. After treatment with indicated concentrations of CH004 in the absence (**e**) or presence (**f**) of the ferroptosis inhibitor deferoxamine for 24 h, HepG2 cells were stained with 2 μM BODIPY^®^ 581/591 C11, a lipid ROS tracker, before the analysis with flow cytometry (Materials and Methods). The FACS histogram plot for green fluorescent (530 nm) was shown in **e** or **f**, and the quantitative data was normalized with the control (DMSO, 100%) and shown in **g**. The ratio between green fluorescence (oxidized BODIPY^®^ 581/591 C11) and red fluorescence (reduced BODIPY^®^ 581/591 C11) was additionally displayed (**e**, **f**). Means ± SDs (*n* = 3). **h** CH004 dose-dependently depletes the intracellular GSH in HepG2 cells. HepG2 cells were seeded in a 6-well culture plate for 24 h before the treatment of CH004 at the indicated concentration for 24 h. Then, the cells from two wells were lysed with 100 μl, 50 mM MES buffer containing 1 mM EDTA (pH 6–7) before the deproteination, and the intracellular total GSH was measured with a commercial glutathione assay kit (Cayman, see “Materials and Methods” section for detailed protocol). The amount of total GSH was normalized with the corresponding protein concentration (BCA protein assay reagent kit, Pierce) and displayed as a percentage of control (DMSO, 100%). Means ± SDs (*n* = 3). Statistical analyses were performed on the raw data by one-way ANOVA with Bonferroni post-tests. ***p < 0.001. All the experiments were independently repeated twice and one representative result is present; *n*.s. no significance

Interestingly, we found the cell viability of HepG2 cells treated with 10 μM CH004 can be dose-dependently recovered by ferrostatin-1, a known ferroptosis inhibitor^15^, but not by the apoptosis inhibitor Z-VAD-FMK or necroptosis inhibitor necrostatin-1 (Fig. 6a–c). Indeed, deferoxamine, an iron chelator, could increase the quantities of viable HepG2 cells by ~ 23% and 35% in the presence of 10 and 15 μM CH004 (Fig. 6d and Supplementary Figure 14a–b), respectively, as assessed by flow cytometry^44^. Similarly, 2 μM ferrostatin-1 could also increase the viable cells from 30.8% to 76.1% in the presence of 15 μM CH004. Moreover, the lipid peroxidation (lipid ROS; ref. ^45^^,^^46^), was also dose-dependently increased with the concentration of CH004 (Fig. 6e, g). 10 μM CH004 caused lipid ROS with an increase of ~ 250%, which could be almost completely reduced by 500 μM deferoxamine (Fig. 6f, g). Consistently, CH004 was found to dose-dependently deplete the intracellular pool of glutathione (GSH) in HepG2 cells (Fig. 6h), a downstream effector for ferroptosis^16^. Also, the level of CTH, the product of CBS and a central metabolite of the transsulfuration pathway^20,47^, was found to be largely reduced (Supplementary Figure 14c), indicating that the activity of CBS or transsulfuration pathway is inhibited upon the treatment of CH004. These data demonstrate that HepG2 cells underwent ferroptosis once the activity of CBS was largely reduced.

### CH004 suppresses in vivo tumor growth

**Fig. 7:**
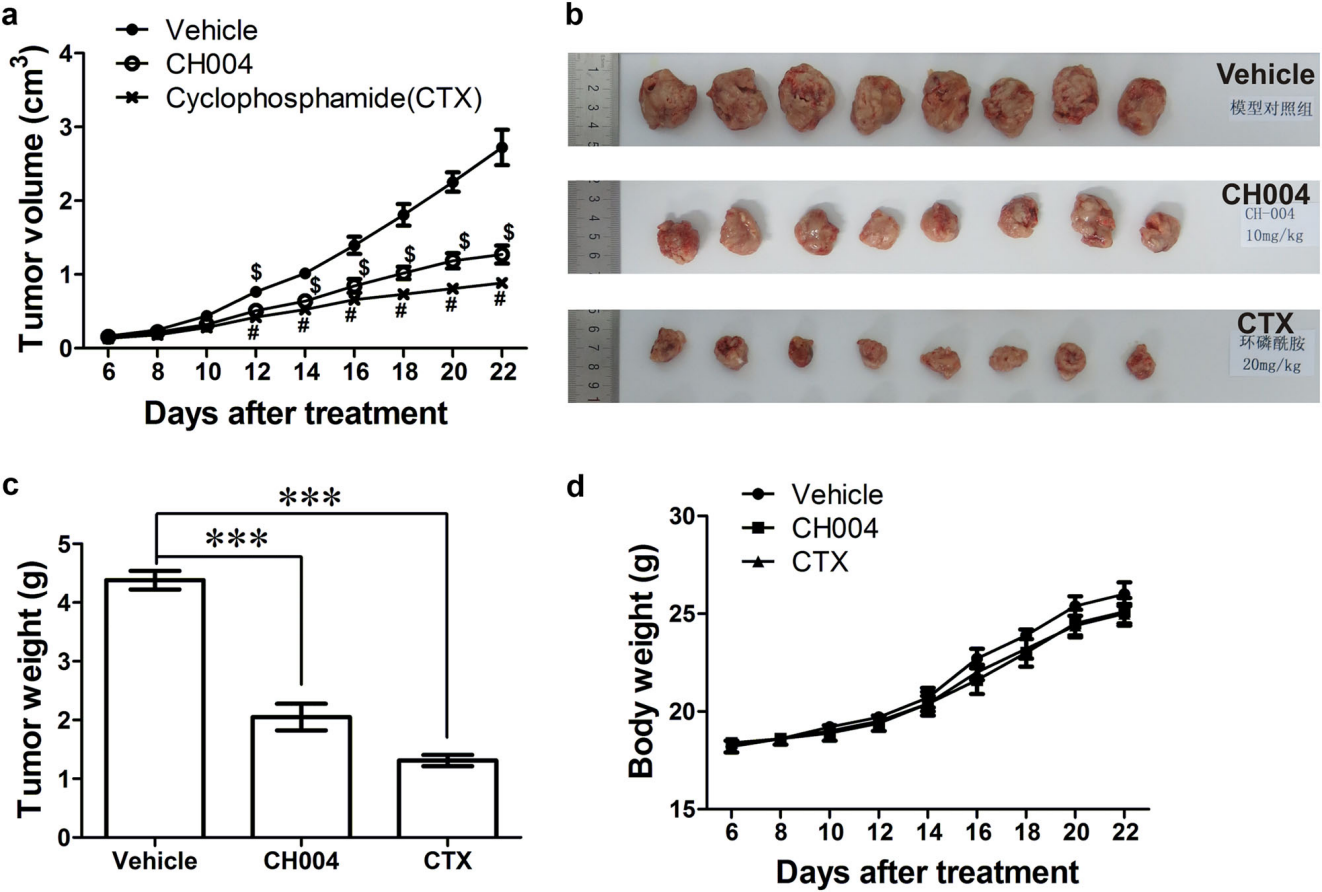
CH004 suppresses tumor growth in vivo. **a**–**c** CH004 reduces the growth rate of tumors in liver tumor xenograft mice model. Mice bearing liver tumor xenografts were treated with CH004 (10 mg/kg/d), cyclophophamide (CTX, 20 mg/kg/d) or PBS (Vehicle) for 21 days. The tumor volumes were measured every two days during day 6 to day 22 and the data was presented in **a** (*n* = 8). The tumor was weighted and recorded after scarification of the mice at the day 22, the tumor specimen were shown in (**b**) and the quantitative data for tumor weight were present in **c** (*n* = 8). **d** CH004 hardly affects the body weight of mice. Mice body weight was measured along with the measurement of tumor volume (*n* = 8). Means ± SDs. Statistical analyses were performed on the raw data by one-way (**c**) or two-way ANOVA (**a**) with Bonferroni post-tests. The significance of the CH004-treated group compared to Vehicle was indicated with $ (**a**), whereas the comparison of the CTX group to Vehicle was with # (**a**). $, # or ***p < 0.001

CH004 (10 mg/kg/day) significantly reduced the growth rate of tumor cells in liver tumor xenograft mice model since the day 12 of treatment (*p* < 0.001; Fig. 7a), an inhibitory effect that is comparable to the treatment of CTX (20 mg/kg/day), a US Food and Drug Administration (FDA)-approved cytotoxic anticancer drug and also known as Cytoxan^48^. Strikingly, CH004 as well as CTX reduced more than half of the tumor volume or weight after a 21-day treatment (Fig. 7b, c), and did not substantially affect the total body weight (Fig. 7d), implying that CH004 at the applied dose is effective and tolerant in mice.

## Discussion

In this study, we identified that CH004 is an effective inhibitor for CBS with an IC_50_ of ~1 μM in the in vitro purified enzyme assay. The efficacy of CH004 in reducing the production of H_2_S in cells was found to be higher than HA or AOAA, two well-known and unspecific inhibitors for hCBS (Fig. 3b, c, Supplementary Figures 7–9)^29^. Additionally, CH004 selectivity inhibits hCBS rather than hCSE in the in vitro purified enzyme assay and in cells at low micromolar concentrations, and shows an efficacy to boost the level of Hcys in cells or rat plasma (Fig. 1, Supplementary Table 1, Figs. 2 and 3). Altogether, these data suggest that CH004 is a potent and bioactive inhibitor for CBS in vitro, in cells and in vivo.

We observed that the genetic ablation of CBS could counter back the inhibition of CH004 on the cell viability, which seems to due to that the knock-down of CBS induces the redox adaptation, a phenomenon leading to enhanced anti-oxidant capacity or amplifying of other anti-oxidant pathways^20,49^. This phenomenon has been observed in the CARS knock-down cells, a gene which is required for the erastin-induced ferroptosis^20^. Knock-down of CARS inhibits the induction effect of erastin on ferroptotic cell death via upregulation of transsulfuration pathway. Also, a similar observation has been obtained for the peroxiredoxin I inhibitor AMRI-59, and both the knock-down and overexpression of the peroxiredoxin I countered back the inhibition of the inhibitor AMRI-59 on cell proliferation^50^. In a consistence with this observation, the expression of CBS wt or CH004-insensetive mutant CBS Q222A was found to partially reverse the inhibitory effects of CH004 on cells (Fig. 4b and Supplementary Figure 15a). Altogether, it suggests that the inhibition of cell proliferation exerted by CH004 is likely dependent on the activity of CBS and the accumulation of ROS.

In this study, we report that the inhibition of CBS caused by CH004 increases the lipid ROS, an indicator for ferroptosis, along with decreasing the viability of tumor cells (Figs. 4a, c, d and 6e, g). In supporting the former, the elevated lipid ROS and cell viability could be significantly counteracted by deferoxamine, an iron chelator and ferroptosis inhibitor (Fig. 6d, f and g). In a similar to the effects and mechanism in the human liver carcinoma cells, CH004 triggers the ferroptosis and the accumulation of lipid ROS in mouse embryonic fibroblasts (MEF, Supplementary Figure 15b), a non-tumor cell line. The potency of CH004 at low micromolar concentrations in the induction of ferroptosis is ∼2-fold in MEF, which seems to be weaker than that of erastin (~3-fold, Supplementary Figure 15c), a well-known ferroptosis inducer^15^. However, erastin at a concentration up to 15 μM or 20 μM did not trigger the apparent accumulation of lipid ROS or cell death in HepG2 cells (Supplementary Figure 15d–e), which was also observed previously and due to the compensatory effects of GSH^44^. The drastic difference between CH004 and erastin on inducing ferroptosis in HepG2 cells suggests that CH004 triggers the ferroptosis via a different molecular mechanism from simply targeting system *X*_c_^-^, which is the target of erastin. The downstream transsulfuration pathway is possible to be another target besides/except interfering with the uptake of cystin. Furthermore, the phenomenon of lipid peroxidation was also observed in CBS-deficient mice^51^. Altogether, these data imply that the transsulfuration pathway mediated by CBS may interact with the homeostasis of Fe^2+^ to disrupt cellular lipid peroxidation, and thus negatively regulates ferroptosis. Interestingly, a recent report indeed observed that knockout of CBS in mice could cause iron-overload and damage in liver^52^.

Apart from the induction of ferroptosis, CH004 also causes HepG2 cells to undergo other cell death forms, which are independent from ferroptosis, apoptosis or necroptosis, since substantial amount of dead cells can’t be rescued with the inhibitors of these pathways (Fig. 6a–d and Supplementary Figure 15e). These results suggest that CH004 triggers the cell death not only via the well-established ferroptotic pathway but also via other unknown mechanism. However, considering of the known ferroptosis inhibitor erastin can’t trigger the ferroptosis in liver carcinoma HepG2 cells (Supplementary Figure 15d-e), the special property of CH004 on the induction of ferroptosis in HepG2 cells seem to offer an alternative chemical tool for triggering ferroptosis.

In conclusion, we designed and synthesized a novel, potent and bioactive inhibitor for hCBS. Importantly, we found the inhibition of CBS could lead to ferroptotic cancer cell death and the new CBS inhibitor effectively reduces the tumor growth in a liver tumor xenograft mice model. The new, potent and bioactive inhibitor could serve as a basis for developing drug leads for the treatment of liver cancer. Further studies on the cross talk between CBS and ferroptotic process in cells is necessary to elucidate the explicit role and underlying molecular mechanisms of transsulfuration pathway in the death of cancer cells.

## Materials and methods

### Plasmids

The truncated hCBS△414–551 (hCBS-413), human CBS or hCSE full-length (CBS-FL or CSE) was subcloned into pGEX-KG, pcDNA3 or pCDH vector using appropriate restriction sites as described^30^. The vectors carrying hCBS mutations were constructed made by site-directed mutagenesis according to Strategene’s instructions using KOD-plus (TOYOBO, Osaka, Japan) and Dpn I (NEB, Ipswich, MA, USA) (see Supplementary Table 3 for details).

### Expression and purification

The GST-tagged hCBS-413, hCBS-413 mutants (T146A, S147A, Q222A or Y223F) or hCBS-FL as well as hCSE were expressed in *E. coli* BL21 and purified by GSH-coupled affinity agarose (for detailed procedures, see ref. ^30^). hDDC was purified by Ni^2+^-coupled affinity column according to the procedures as described previously^36^.

### IC_50_ determination

The IC_50_ values of CH004 for hCBS-413, hCBS-413 mutants, hCBS-FL, hCSE or hDDC were determined according to the standard assay conditions otherwise indicated^30^.

### Quantification of H_2_S by methylene blue method

The amount of H_2_S in the in vitro assay or rat plasma was determinate according to the method reported by Stipanuk and Beck^53^.

### H_2_S-donor interfering assay

To exclude the possibility that CH004 reacts with the H_2_S during the assay, a counterscreen assay was constructed based on a previously described protocol by using NaSH (ACROS, Geel, Belgium)^33^, a commonly-used H_2_S donor. Briefly, 1 μL CH004 at indicated concentrations was added together with 100 μM NaHS (final concentration) into the reaction well of the tandem-well plate, which contains only the assay buffer. 5,5′-Dithiobis(2-nitrobenzoic acid) (DTNB; Sangon, Shanghai, China) was then added into the coupled detection well before an immediate seal of the plate. The sealed assay plate was incubated for 50 min at 37 °C before the absorbance at 413 nm was measured.

### Surface plasmon resonance assays

Surface Plasmon Resonance assays (SPR) with a BIAcore T200 (GE Healthcare, Uppsala, Sweden) were used to observe the direct interaction between inhibitors and hCBS. The SPR assay was performed in running buffer (1× PBS with 0.05% P20) and the purified GST-tagged CBS-413 (500 μg/mL) was immobilized onto a flow cell of a CM5 sensor chip using a GST antibody coupling kit in running buffer. The K_D_ values were determined with the Biacore evaluation 3.1 software.

### Cell culture

HepG2 cells were maintained in MEM (Gibco, Gaithersburg, MD, USA) supplemented with 1× non-essential amino acids (NEAA; Gibco), 10% fetal bovine serum (FBS; Gibco), and 1% (w/v) penicillin and streptomycin (P/S; Gibco, 10378016) in a humidified 5% CO_2_ atmosphere at 37 °C. HEK293T, MDA-MB-231, Panc-28, Huh7 or MEF cells were maintained in DMEM (Gibco) in the presence of 10% FBS and 1% P/S. HCT116 cells were maintained in McCoY’S 5 A medium (Gibco) in the presence of 10% FBS and 1% P/S. H22 cells were maintained in RPMI-1640 (Gibco) in the presence of 10% FBS and 1% P/S.

### Stable cell lines

HEK293T cells stably expressing hCBS-FL WT or Q222A mutant were generated using lentiviral particles carrying pCDH-hCBS or pCDH empty vector (EV) and pPACK Packaging Plasmid Mix (SBI, Mountain View, CA, USA) according to the manufacturer’s instructions. Similarly, CBS-knock-down HEK293T cells or control cells were obtained using the miRzip lentiviral vector according to the manufacturer’s protocol (SBI, MZIPxxxPA-1). The infected cells were then incubated with 2.5 μg/mL puromycin for two weeks until stable clones were obtained. The target sequence of shRNA-CBS was 5′-GTAGTTCCGCACTGAGTCG-3′, which has been used previously^54^. The scramble control was 5′-TCCGCAGGTATGCACGCGT-3′.

### Cell viability

To measure cell viability and cytotoxicity, HepG2, HEK293T, Huh-7, H22, Panc-28, HCT116 and MDA-MB-231 cells were seeded at a density of ~ 2 × 10^4^ cells per well in 96-well plates (Coring, NY, USA) for one day, followed by incubation with DMSO or various concentrations of tested compound for indicated times. Then, the cells were collected to measure the cell viability using the CellTiter 96^®^ Aqueous One Solution Cell Proliferation Assay (Promega, G3581) according to the standard protocols.

### Western blotting

Cells were collected, lysed in Glo^®^ lysis buffer (Promega, Madison, Wisconsin, USA) and the cell supernatant was obtained after centrifugation, followed by being separated on 10% SDS-PAGE gels [protein normalized by using the BCA protein assay reagent kit (Pierce, Waltham, MA, USA)], transferred onto PVDF membrane (Whatman, Dassel, Germany) and probed with an anti-CBS antibody (3E1, 1:2000; Abnova, Walnut, CA, USA), anti-CSE antibody (1:500, GeneTex, San Antonio, TX, USA) or an antibody for GAPDH (1:2000, CST, Boston, MA, USA).

### Detection of H_2_S in living cells with fluorescent probe

H_2_S levels in living cells were detected using a H_2_S-specific fluorescent probe according to a protocol described previously^31,55^. To measure H_2_S in transiently transfected HEK293T cells, the cells were seeded on poly-d-Lys-coated coverslips (Thermo Scientific, Waltham, MA, USA) in 12-well plates at a density of ~ 1 × 10^5^ cells per well, and hCBS or hCSE plasmids were transiently transfected by X-tremeGENE HP DNA Transfection Reagent according to the manufacturer’s protocol (Roche, Basel, Switzerland). After 24 h, the transfected cells were treated with DMSO or inhibitors for another 8 h, followed by staining with 7-azide-4-methylcoumarin (AzMC; Sigma-Aldrich). Similarly, HEK293T cells stably expressing CBS WT, Q222A mutant or EV were seeded on poly-d-Lys-coated coverslips in 12-well plates for one day before treatment with DMSO or inhibitors for 8 h. After incubation with 50 μM AzMC at 37 °C for 30 min, the cells were washed 4 times with PBS, and the fluorescent images were recorded with excitation by 405 nm laser by using a A1Si (Nikon) confocal microscope. The intensity of the blue fluorescent signals, indicating the amount of H_2_S, was quantified by ImageJ software (NIH, Bethesda, ML). The H_2_S signal was then corrected for the background signal detected in HEK293T cells transiently or stably expressing of empty vector (HEK293T-EV) and normalized by the corresponding cell-occupying area and compared with the control (DMSO group, 100%). Similarly, the H_2_S signal in HepG2 cells was quantified by AzMC staining, corrected for the background signal outside the cells, normalized by the cell area and expressed as percentages of DMSO control (100%).

### Quantification of homocysteine

HepG2 or HEK293T cells were seeded in poly-d-Lys-coated 6-well plates at a density of ∼ 5 × 10^5^ cells per well for one day, treated with DMSO or various concentrations of compounds for another 12 h before the collection by scraping or trypsinization. The collected cells were suspended in 50 μL PBS and lysed by freezing and thawing (see above), the 35 μL supernatant was then used to measure the content of homocysteine (Hcys) according to the manufacturer’s procedure (IBL, Gunma, Japan).

### Subcellular localization of hCBS and H_2_S

HEK293T or HepG2 cells were seeded on coverslips in 12-well plates. After reaching ~ 70% confluence, the cells were incubated with 200 nM Mito-Tracker^®^ Green (Invitrogen, Carlsbad, CA, USA) for 30 min at 37 °C in PBS, fixed with 4% PFA, permeabilized with 0.1% Triton X-100 in 1% BSA-PBS, and detected with anti-CBS antibody (3E1, 1:200, Abnova) or anti-CBS antibody (A-2, 1:200; Santa Cruz Biotechnology Inc., Santa Cruz, CA, USA) before staining with Alexa Fluor 594-labelled donkey anti-mouse secondary antibody (1:400, Jackson ImmunoRearch, West Grove, PA, USA) and DAPI (Sigma-Aldrich). The fluorescent images were captured using an A1Si confocal microscope (Nikon, Japan).

To elucidate the subcellular localization of H_2_S, HepG2 cells were seeded on poly-D-Lys-coated coverslips in 12-well plates. After treatment with DMSO or inhibitors for 8 h, cells were sequentially incubated with 200 nM Mito-Tracker^®^ Green and 50 μM AzMC for 30 min at 37 °C in PBS, and then fluorescent images were taken using a confocal microscope.

### Quantitative analysis of cellular cystathionine via LC/ESI-MS/MS

The effect of CH004 on the level of CTH in HepG2 cells was determined by a liquid chromatography-tandem mass spectrometry (LC/ESI-MS/MS). We followed a condition, which was previously established with LC/ESI-MS/MS^56^, for quantification of CTH. The singly charged parent ion for CTH was observed at 223.1 m/z. The product ion scan showed dominant product ions at m/z values of 134 and 88.1. We selected the 223.1 → 88.1 transition for the quantitative method. Briefly, after the incubation with the compounds for 8 h in the presence of 100 μM Hcys and 100 μM Ser, the HepG2 cells were lysed with liquid nitrogen. 10 μl supernatant was injected to the Ultra Performance Liquid Chromatographic system on a Zorbax XDB C18 reverse phase column (4.6 × 150 mm, temperature-controlled at 10 °C), and eluted isogradiently with a solution mixed from acetonitrile (in the presence of 0.1% formic acid) and water (0.1% formic acid) (2:98, v/v) at a flow rate of 0.4 ml/min in a diode array detector (Agilent G4212A). The Agilent 6470 UHPLC-QqQ/MS system composes of a chomatographic system (Agilent 1260 Infinity) and a Triple Quad mass spectrometer fitted with an ESI source. Data was acquired in the MRM mode using Agilent MassHunter Workstation Data Acquisition Software (revision B.04).

### Flow cytometric analysis of cell cycle, cell death, cellular ROS and lipid ROS

HEK293T, 293T-shNT or 293T-shCBS cells were seeded in 6-well plates at a density of ~ 4 × 10^5^ cells per well for one day, then incubated with DMSO or inhibitors for 12 h before collection by trypsinization. After being washed once with PBS, the cells were fixed in ice-cold 75% ethanol overnight. The cells were then washed once and stained with propidium iodide (PI, 20 μg/mL in PBS buffer containing 0.1% Triton X-100 and 0.1 mg/mL DNase-free RNaseA, final concentrations) at 37 °C for 30 min before analysis using an LSR Fortessa flow cytometer (BD Bioscience, Franklin Lakes, New Jersey, USA), and the data were analyzed using the ModFit LT 3.2 software (Verity Software House, Topsham, ME, USA).

To characterize the cell death triggered by CH004, endogenous HEK293T or HepG2 cells were seeded in 6-well plates at a density of ~ 4 × 10^5^ cells per well for one day, then incubated with indicated concentrations of CH004 for an additional 12 h before being collected to analyze the dying cells using a FITC Annexin V Apoptosis Detection Kit according to the manufacturer’s protocol (BD Pharmingen, San Diego, CA, USA).

To investigate the intracellular ROS levels, HEK293T or HepG2 cells were seeded in 6-well plates for one day, then incubated with the indicated inhibitors and 1 mM H_2_O_2_ for 12 h and 30 min, respectively. After trypsinization and one wash in PBS, the collected cells were resuspended in PBS and incubated with 100 μM probe DCFH-DA for 30 min at 37 °C before being analyzed using an LSR Fortessa flow cytometer.

To detect lipid peroxidation upon treatment with CH004, HepG2 cells or mouse embryonic fibroblasts (MEF) were seeded in 12-well plates with ~ 1.8 × 10^5^ cells per well for 24 h. After treatment with the inhibitor for another 24 h, cells were stained with BODIPY^®^ 581/591 C11 (Invitrogen) for 1 h at a final concentration of 2 μM at 37 °C^57^. The cells were then resuspended in an appropriate amount of PBS after being washed once with PBS. Lipid peroxidation, i.e., lipid ROS, was analyzed by detection of the fluorescence using an LSR Fortessa flow cytometer. The data were recorded at 530/30 nm (green fluorescent) and 670/30 nm (red fluorescent) wavelengths after excitation at 488 nm. A minimum of 20000 events was used for the analysis. All the experiments were independently repeated twice in triplicate.

### Measurement of intracellular glutathione

HepG2 cells were seeded in a 6-well culture plate for 24 h before the treatment of CH004 at the indicated concentration for 24 h. Then, the cells from two wells were collected by cell scraper and washed once with PBS. The intracellular total glutathione (GSH) was measured with a commercial glutathione assay kit under the standard protocol (Cayman Chemical, Ann Arbor, USA). Briefly, the cell pellet was resuspended using 100 μl 50 mM MES buffer containing 1 mM EDTA (pH 6–7) and lysed with liquid nitrogen. Supernatant was then collected after centrifugation at 12,000 × *g* for 15 min at 4 °C before the deproteination by adding an equal volume of 2.5 M metaphosphoric (MPA, Sigma). After the incubation for 5 min, the mixture was centrifuged at 3000 × *g* for 5 min. The supernatant was collected and added appropriate amount of 4 M triethanolamine (Sigma, 50 μl per 1 ml of the supernatant). The deproteinated and neutralized supernatant was used for the determination of the amount of total GSH by incubating with the assay reagents (from the kit) in a 96-well plate. After an incubation of 25 min, the absorbance at 405 nm was recorded and converted into the amount of GSH with a standard curve.

### Hemorrhagic shock rat model

A known hemorrhagic shock rat model was employed to assess the blood pressure-restoration effect of CH004 and PAG^41^, a CSE inhibitor. All the procedures were approved by the Animal Care and Use Committee at Shanghai Jiao Tong University.

### Liver tumor xenograft mice model

The in vivo effect of CH004 on tumor growth was evaluated in a xenograft mice model of liver cancer. The experiment was independently performed by Jiangsu KeyGEN BioTECH Co. LTD (Jiangsu, China) and followed the standard procedures that were established and approved by the local Animal Care and Use Committee. Briefly, H22 mouse liver tumor cells were intraperitoneal injected into female ICR mice (SLAC Laboratory Animal Co. Ltd, Shanghai, China). 8 days later, mice with distended abdomen were killed, and the ascites was collected, washed and resuspended with PBS. 0.1 ml cell suspension with a density of 1 × 10^7^/ml was subcutaneously injected into the right dorsum of ICR female mice (~20 g). The mice bearing H22 tumor cells were then randomized into groups (*n* = 8) on the following day after the implantation. Then, the mice were injected via a tail vein (i.v.) with PBS, CH004 (10 mg/kg) or cyclophosphamide (CTX, a known anticancer drug; 20 mg/kg) once per day and for 21 days. Tumor sizes were measured every 2 days starting from the day 6 using a caliper, and tumor volume was calculated using the formula *V* *=* length × width^2^ × *π* / 6. The body weights of mice were recorded under the same conditions. The mice were sacrificed on day 22, and the tumors were taken out and weighted.

### Statistical analysis

Statistical analysis was performed on the raw data for each group by one-way or two-way ANOVA. A *p* < 0.05 was considered statistically significant.

## Electronic supplementary material

Supplementary Information
