# Supplementary material for: A pharmacological probe identifies cystathionine β-synthase as a new negative regulator for ferroptosis
Source: Cell Death Dis. 2018 Sep 26;9(10):1005. doi: 10.1038/s41419-018-1063-2 (PMC6158189; doi:10.1038/s41419-018-1063-2)
Supplement: Supplementary file 1 — Supplementary Information [file 41419_2018_1063_MOESM1_ESM.doc]

Supplementary information

**A pharmacological probe identifies cystathionine β-synthase as a new negative regulator for ferroptosis**

Li Wang^1,*^, Hao Cai^2,*^, Youtian Hu^1^, Fan Liu^1,3^, Shengshuo Huang^3^, Yueyang Zhou^1^, Jing Yu^3^, Jinyi Xu^2^, Fang Wu^1^

^1^Key Laboratory of Systems Biomedicine (Ministry of Education), Shanghai Center for Systems Biomedicine, Shanghai Jiao Tong University, Shanghai, China

^2^State Key Laboratory of Natural Medicines and Department of Medicinal Chemistry, China Pharmaceutical University, Nanjing, China

^3^State Key Laboratory of Microbial Metabolism, Sheng Yushou Center of Cell Biology and Immunology, School of Life Sciences & Biotechnology, Shanghai Jiao Tong University, Shanghai, China

^*^These authors contributed equally to this work.

Running title: Inhibition of CBS triggers ferroptosis

**Table of Contents**

MATERIALS AND METHODS

[**S4**](#__RefHeading___Toc482303187)

[Scheme S1. Synthesis of CH004 S](#__RefHeading___Toc482303188)7

[Figure S1. MS and NMR spectras of CH004 S](#__RefHeading___Toc482303189)8

[Figure S2. The mode of action of CH004 in inhibiting hCBS S](#__RefHeading___Toc482303194)12

[Figure S3. Enzyme kinetics of hCBS mutants and H_2_S imaging in HEK293T cells transfected with empty vector S](#__RefHeading___Toc482303195)14

[Figure S4. Visualization of H_2_S in HEK293T cells expressing of hCBS or hCSE by AzMC probe under confocal microscope S](#__RefHeading___Toc482303196)16

[Figure S5. CH004 dose-dependently decreased the H_2_S level in HEK293T cells overexpressing hCBS WT S18](#__RefHeading___Toc482303197)

[Figure S6. CH004 hardly affected the H_2_S level in HEK293T cells expressing hCBS Q222A mutant S19](#__RefHeading___Toc482303199)

[Figure S7. The CH004-insensitive CBS Q222A mutant can be inhibited by HA S20](#__RefHeading___Toc482303200)

[Figure S8. The effect of AOAA on the level of H_2_S in HEK293T cells expressing hCBS WT S](#__RefHeading___Toc482303201)22

[Figure S9. The effect of AOAA on the level of endogenous H_2_S in HepG2 cells S](#__RefHeading___Toc482303202)24

Figure S10[. CH004 didn’t restore the blood pressure in the rat hemorrhagic shock model S](#__RefHeading___Toc484253917)26

Figure S11. The effects of CH004 on cell viability in various types of cancer cell lines S27

[Figure S12. CH004 inhibits the proliferation of cells via specifically targeting hCBS S29](#__RefHeading___Toc482303204)

[Figure S13. hCBS co-localizes with mitochondria in HepG2 and HEK293T cells. S31](#__RefHeading___Toc482303205)

[Figure S14. CH004-induced cell death was prevented by ferroptosis inhibitors and CH004 reduces the intracellular amount of cystathionine in HepG2 cells. S](#__RefHeading___Toc482303206)32

Figure S15. The mode of actions for CH004 in cells. S34

[Table S1. The inhibitory effects of CH004 on purified hCBS, hCSE and hDDC. S](#__RefHeading___Toc484253917)36

[Table S2. The inhibitory effects of CBS inhibitors on hCBS WT and mutants S](#__RefHeading___Toc484253917)37

[Table S3. Primer sequence S](#__RefHeading___Toc482303208)38

**References** [**S**](#__RefHeading___Toc482303208)**39**

**MATERIALS AND METHODS**

**Materials**

5,5’-Dithiobis(2-nitrobenzoic acid) (DTNB), hydroxylamine hydrochloride (HA), glutathione (reduced form), N,N-dimethyl-p-phenylenediamine-sulfate, zinc acetate (ZnAC), trichloroacetic acid (TCA), iron chloride (FeCl_3_), paraformaldehyde (PFA), isopropyl-β-d-thiogalactopyranoside (IPTG), Triton X-100, l-cysteine (L-Cys) and l-serine (L-Ser) were purchased from Sangon (Shanghai, China). GST agarose was purchased from Aogma (Shanghai, China). d,l-homocysteine (Hcys), d,l-propargylglycine (PAG), pyridoxal 5’-phosphate hydrate (PLP), aminooxyacetic acid (AOAA), poly-D-lysine hydrobromide (poly-D-Lys), s-(5’-adenosyl)-l-methionine iodide (SAM), DMSO, propidium iodide, ferrostatin-1, 2’,7’-dichlorofluorescein diacetate (DCFH-DA) and crystal violet were purchased from Sigma-Aldrich (Steinheim, Germany). Z-VAD-FMK, deferoxamine mesylate and necrostatin-1 were purchased from MedChem Express (Monmouth Junction, NJ). Erastin was brought from Selleck Chemicals (Houston, TX).

General procedures for synthesis

Most of chemicals and solvents were purchased from commercial sources. Further purification and drying were employed by standard methods when necessary. ^1^H NMR and ^13^C NMR spectra were recorded with a Bruker AV-300 spectrometer in the indicated solvents (TMS as internal standard): the values of the chemical shifts are expressed in *δ* values (ppm) and the coupling constants (*J*) in Hz. ESI-MS spectra were recorded on an Agilent1100 LC-MSD-Trap/SL spectrometer and high-resolution mass spectra (HR-ESI-MS) for CH004 were recorded using an Agilent QTOF 6520.

1-methylpyrimidine-2,4,6(1H,3H,5H)-trione (**3**)

To a solution of N-methylurea (**2**, 3.0 g, 40.5 mmol) and malonic acid (**1**, 5.06 g, 48.6 mmol) in 11 ml of acetic acid at 60 ^o^C, 7.8 ml of acetic anhydride was added slowly. After the completion of the addition, the reaction mixture was stirred at 90 ^o^C for 4 h, and then cooled to room temperature. The solvent was removed under reduced pressure, and the product (**3**, 3.77 g, yield 66%) was recrystallized from ethanol. ^1^H NMR (300 M Hz, CDCl_3_): *δ* 8.65 (s, 1H, NH), 3.67 (s, 2H, CH_2_), 3.31 (s, 3H, NCH_3_); ESI-MS *m/z*: 141.0 [M-H]^-^.

6-chloro-3-methylpyrimidine-2,4(1H,3H)-dione (**4**)

Water (0.65 ml) was added dropwise to the suspension of compound **3** (3.5 g, 24.6 mmol) in POCl_3_ (13.8 ml, 147.8 mmol) at 0 ^o^C. The mixture was stirred at 60 ^o^C for 7 h. The excessive phosphorus oxychloride was removed under reduced pressure and the residue was poured into ice-water. The mixture was neutralized with 10% sodium hydroxide, and the precipitate was collected by filtration. The obtained solid was recrystallized from ethanol to give 1.73 g of product as white solid (yield 44%). ESI-MS *m/z*: 159.0 [M-H]^-^.

3-methyl-6-(1’-methylhydrazinyl)pyrimidine-2,4(1H,3H)-dione (**5**)

To the suspension of 6-chloro-3-methyluracil (**4**, 5.0 g, 31 mmol) in 45 ml of ethanol, methylhydrazine sulfate (13 g, 93 mmol) and triethylamine (18 ml, 124 mmol) were added. The mixture was stirred at reflux for 4 h, then cooled to room temperature. The white precipitate was collected by filtration, washed with ethanol and dried to give compound **5** as a white solid (3.49 g, yield 66%). ^1^H NMR (DMSO-*d_6_*): *δ* 9.72 (s, 1H), 4.66 (s, 1H), 3.02 (s, 3H), 2.97 (s, 3H); ^13^C NMR (DMSO-*d_6_*): *δ* 163.38, 154.30, 150.85, 72.74, 26.48; ESI-MS *m/z*: 169.1 [M-H]^-^.

3-methyl-6-(1-methyl-2-(2-methylpropylidene)hydrazinyl)pyrimidine-2,4(1*H*,3*H*)-dione (**6a**)

Isobutyraldehyde (176 μL, 1.94 mmol) was added to the suspension of 3-methyl-6-(1’-methylhydrazinyl)uracil (**5**, 300 mg, 1.76 mmol) in 5 ml ethanol. The mixture was stirred at reflux overnight, and then cooled to room temperature. The yellow precipitate was collected by filtration, washed with ethanol and dried to give compound **6a** as a white solid (160 mg, yield 40%). ESI-MS *m/z*: 223 [M-H]^-^.

6-(2-(cyclopropylmethylene)-1-methylhydrazinyl)-3-methylpyrimidine-2,4(1*H*,3*H*)-dione (**6b**)

Intermediate **6b** was obtained as a white solid (201 mg, 51%) by following the same procedure as for **6a**. ESI-MS *m/z*: 221.1 [M-H]^-^.

3-methyl-6-(1-methyl-2-(2-phenylethylidene)hydrazinyl)pyrimidine-2,4(1*H*,3*H*)-dione (**6c**)

Intermediate **6c** was obtained as a white solid (375 mg, 78%) by following the same procedure as for **6a**. ESI-MS *m/z*: 273.1 [M+H]^+^.

6-(2-(furan-2-ylmethylene)-1-methylhydrazinyl)-3-methylpyrimidine-2,4(1*H*,3*H*)-dione (**6d**)

Intermediate **6d** was obtained as a white solid (247 mg, 56%) by following the same procedure as for **6a**. ESI-MS *m/z*: 247.1 [M-H]^-^.

3-methyl-6-(1-methyl-2-(pyridin-2-ylmethylene)hydrazinyl)pyrimidine-2,4(1*H*,3*H*)-dione (**6e**)

Intermediate **6e** was obtained as a white solid (164 mg, 36%) by following the same procedure as for **6a**. ESI-MS *m/z*: 258.1 [M-H]^-^.

3-methyl-6-(1-methyl-2-(naphthalen-1-ylmethylene)hydrazinyl)pyrimidine-2,4(1*H*,3*H*)-dione (**6f**)

Intermediate **6f** was obtained as a white solid (473 mg, 87%) by following the same procedure as for **6a**. ESI-MS *m/z*: 307.1 [M-H]^-^.

3-benzyl-1,6-dimethylpyrimido[5,4-*e*][1,2,4]triazine-5,7(1*H*,6*H*)-dione (**CH004**)

**CH004** was obtained as a yellow solid (188 mg, 55%) by following the synthetic route as shown in Scheme S1. ^1^H NMR (300 M Hz, Chloroform-*d*): *δ* 7.47-7.04 (m, 5H), 4.22 (s, 2H), 4.03 (s, 3H), 3.40 (s, 3H); ^13^C NMR (75 M Hz, CDCl_3_): *δ* 157.99, 156.73, 153.79, 148.27, 145.04, 135.16, 128.52, 128.29, 126.86, 42.79, 41.37, 28.50; ESI-MS *m/z*: 284.1 [M+H] ^+^.

**
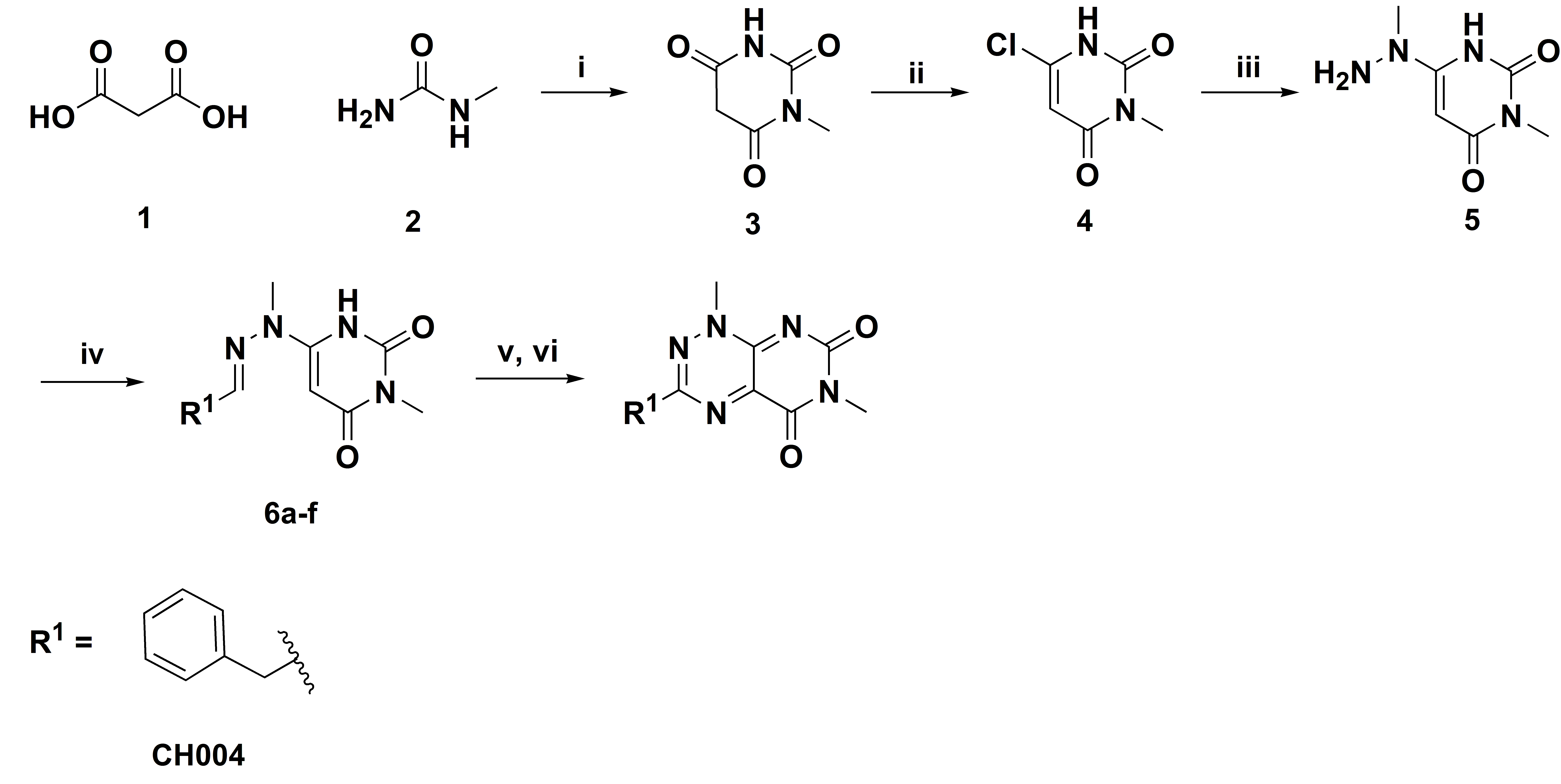
**

Scheme S1. Synthesis of CH004**.** Synthesis scheme of CH004. Reaction conditions: i) Acetic acid, acetic anhydride, 60-90 °C, 4 h; ii) POCl_3_, H_2_O, reflux, 1 h; iii) Methylhydrazine sulfate, TEA, EtOH, reflux, 4 h; iv) R^1^CHO, EtOH, reflux, overnight; v) NaNO_2_, H_2_O, 0-25 °C. 12 h; vi) DTT, EtOH, r.t., 18 h.

**A**


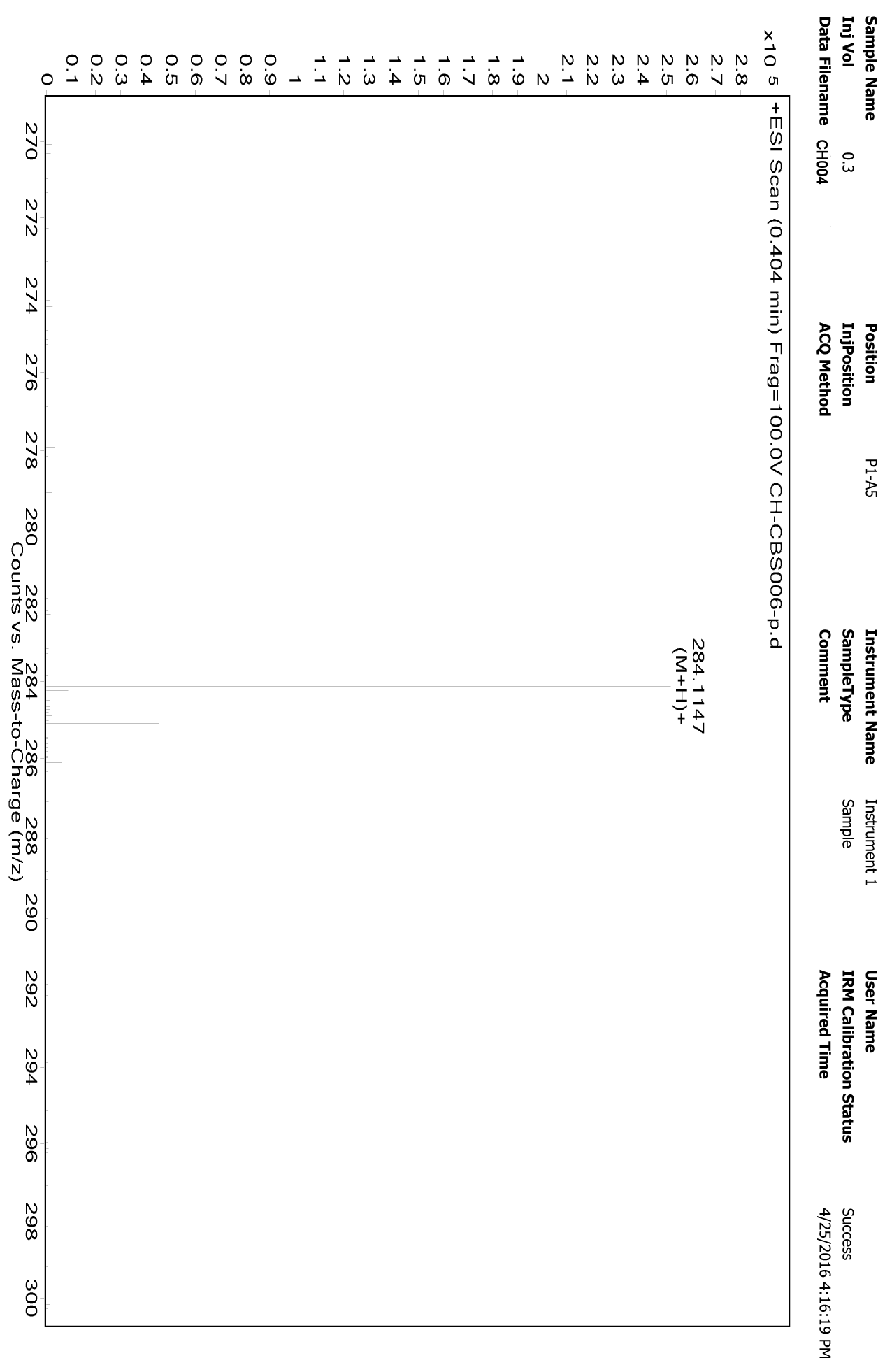


**B**


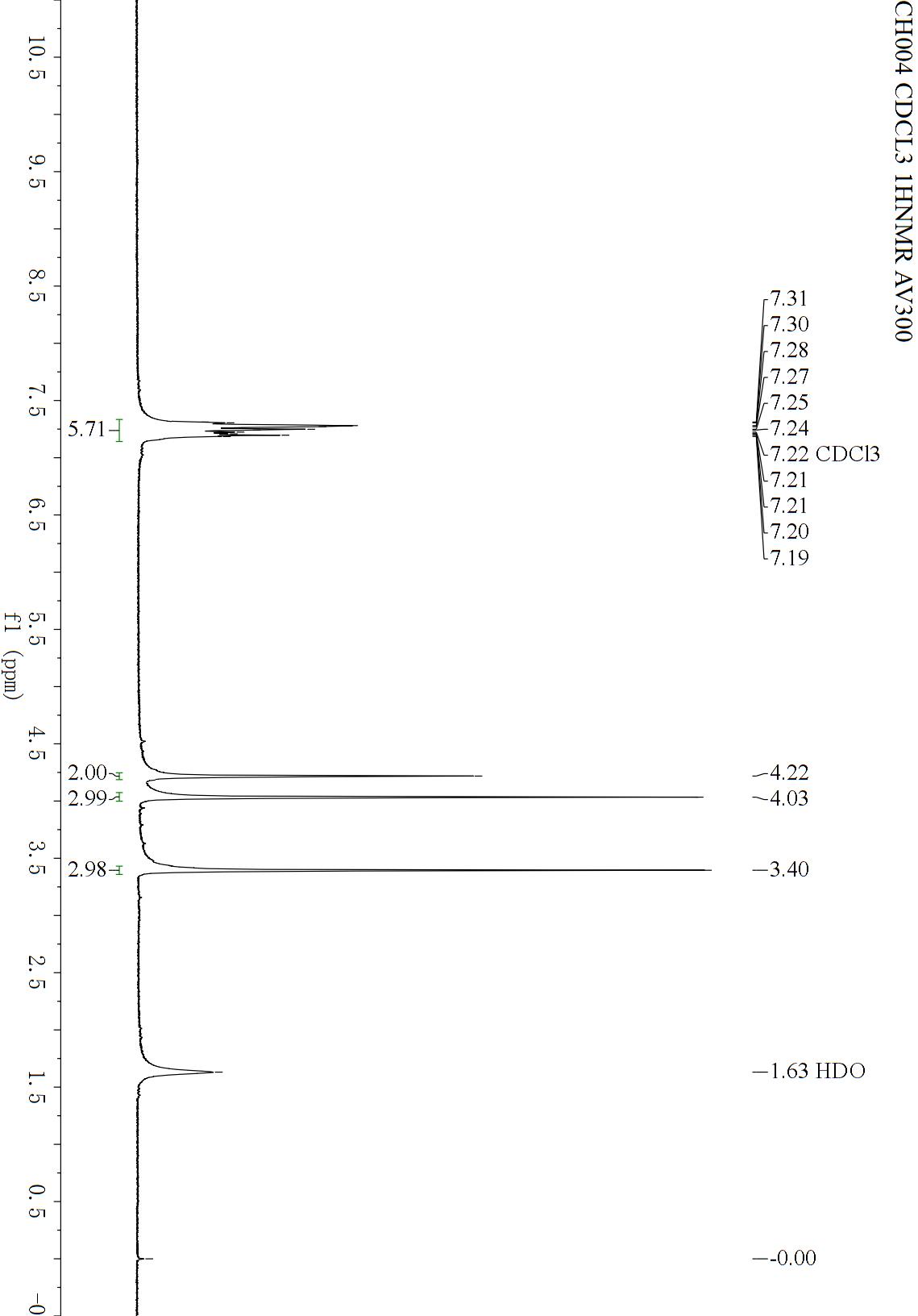


**C**


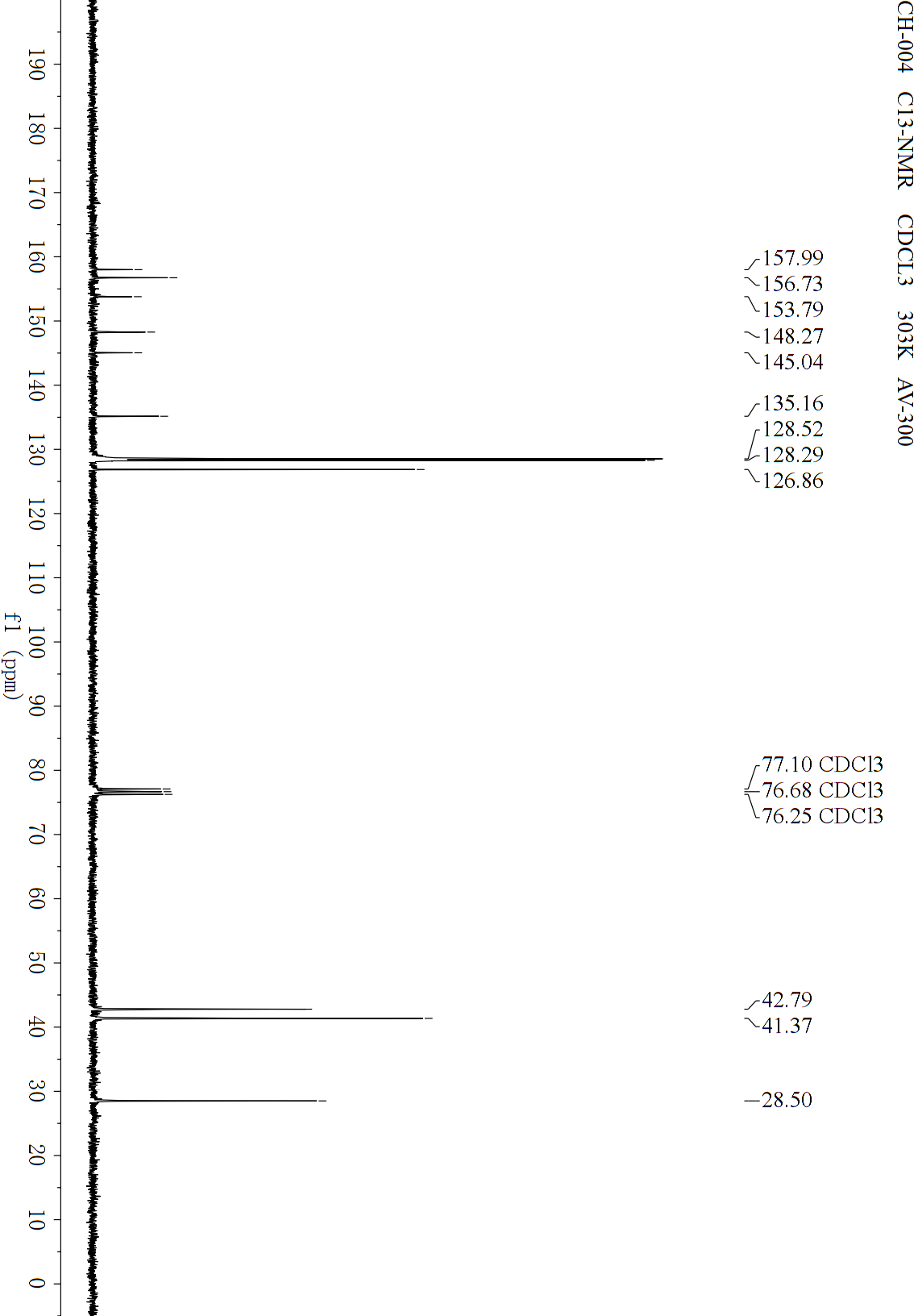


Figure S1. MS and NMR spectras of CH004**.** (**A**) HR-ESI-MS spectra of CH004. The molecular formula of C_14_H_13_N_5_O_2_ was deduced from a [M+H]^+^ ion peak at *m/z* 284.1147 in the HR-ESI-MS, which is consistent with the calculated molecular weight for [M+H], 284.1142. (**B**) ^1^H NMR spectra of CH004. The ^1^H NMR spectrum of CH004 shows the presence of one benzene ring [*δ* 7.47-7.04 (m, 5H)], one methylene [*δ* 4.22 (s, 2H)] and two methyl [*δ* 4.03 (s, 3H), *δ* 3.40 (s, 3H)]. (**C**) ^13^C NMR spectra of CH004 (CDCl_3_, 75 M Hz) *δ* (ppm) 157.99, 156.73, 153.79, 148.27, 145.04, 135.16, 128.52, 128.29, 126.86, 42.79, 41.37, 28.50.


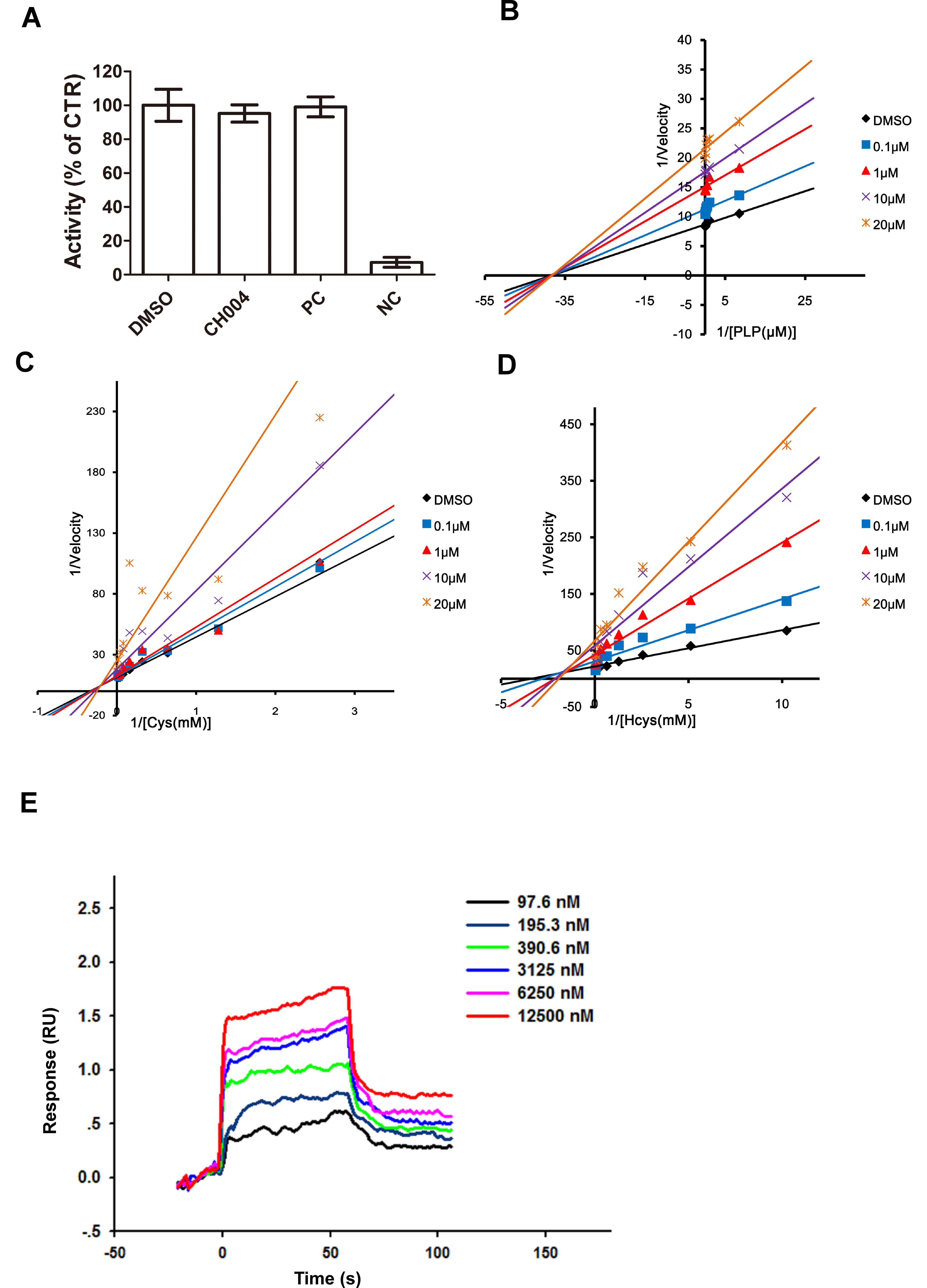


Figure S2. The mode of action of CH004 in inhibiting hCBS**.** (**A**) CH004 reversibly binds to hCBS. After incubation with DMSO or CH004 at 10 μM for 40 min, hCBS (20 μM) was diluted 200-fold and the activity was measured according to the standard assay protocol (Materials and Methods). The data are presented as percentages of control (CTR; DMSO, 100%) and shown as means ± SDs (n = 5). The effects of 0.05 and 10 μM CH004 on hCBS (100 nM) without pre-incubation were defined as positive control (PC) and negative control (NC), respectively. (**B**) Inhibition of hCBS by CH004 as a function of PLP concentration with Cys (20 mM) together with Hcys (10 mM) as substrates (non-competitive inhibition constant αK_i_=15.2 ± 4.9 μM). (**C**) Inhibition of hCBS by CH004 as a function of Cys concentration in the presence of 100 μM PLP and the absence of Hcys (mixed inhibition constant αK_i_=26.4 ± 8 μM). (**D**) Inhibition of hCBS by CH004 as a function of Hcys concentration in the presence of 20 mM Cys and 100 μM PLP (mixed inhibition constant αK_i_’=7.2 ± 1.6 μM). Each point in the Lineweaver-Burk plots represents the means of triplicate. The αK_i_ or αK_i_’ values were calculated by using the non-linear fitting method for enzyme kinetics in GraphPad Prism 5. (**E**) Surface Plasmon Resonance assays analysis of the binding of CH004 to hCBS. Solutions with various concentrations of CH004 (0.097–12.5 μM) were injected into the chamber with a hCBS-coated sensor chip. The change of response units is shown over time. Dissociation constants for CH004 (K_D_) were calculated by using Biacore evaluation software (Materials and Methods).


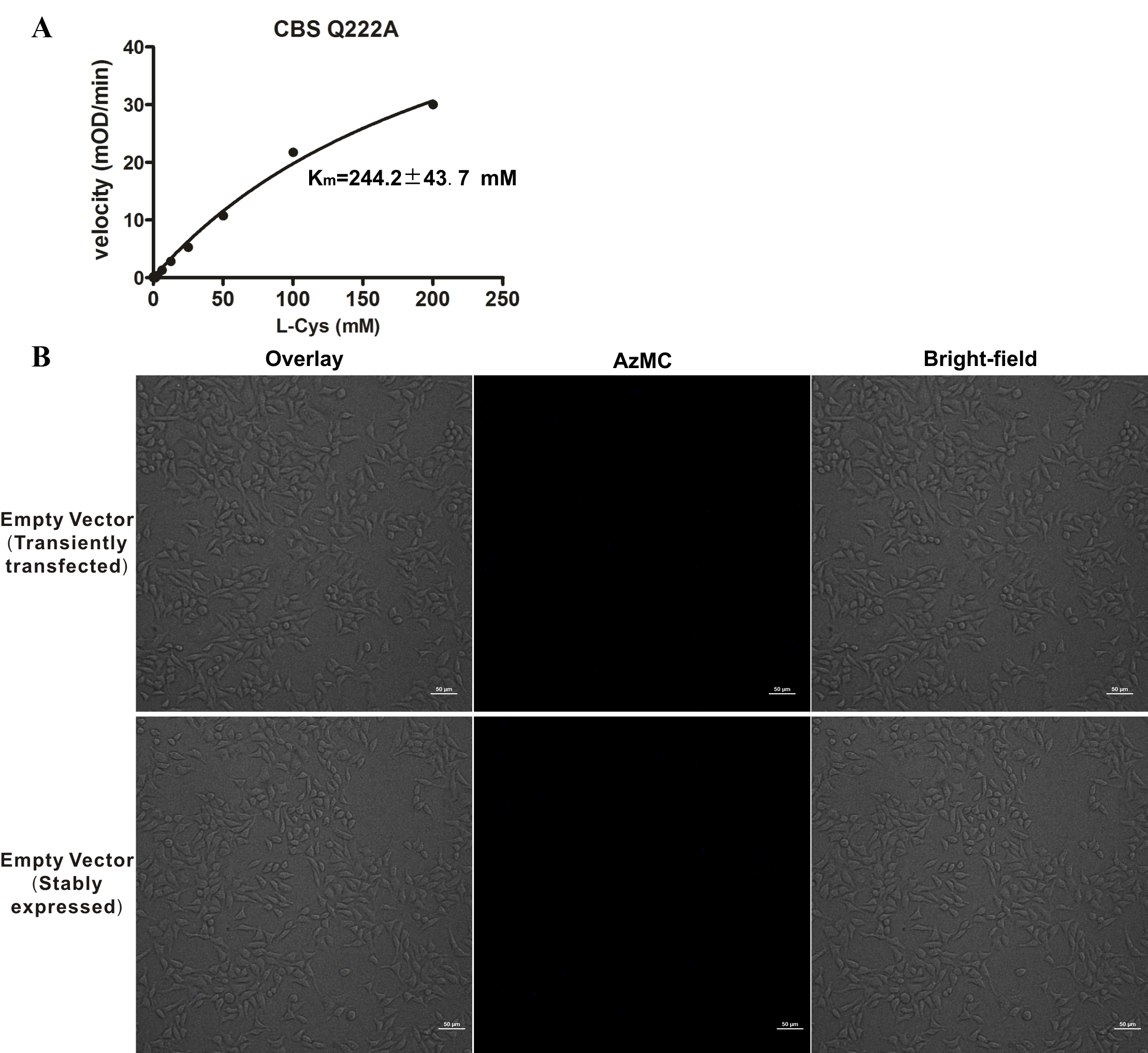


Figure S3. Enzyme kinetics of hCBS mutant and H_2_S imaging in HEK293T cells transfected with empty vector**.** (**A**) Kinetics of CBS Q222A mutant in the presence of various concentrations of Cys (0-200 mM). The activity of purified CBS-FL Q222A enzymes were measured according to the standard assay protocol (Materials and Methods). The K_m_ values were calculated by using the non-linear fitting method for enzyme kinetics in GraphPad Prism 5. (**B**) H_2_S imaging in HEK293T-EV cells. HEK293T cells transiently or stably expressing empty vector (EV) were cultured for 24 h, and followed by staining with 50 μM H_2_S-specific probe AzMC (Materials and Methods), the fluorescent images of H_2_S were taken using a Nikon-A1Si confocal microscope. The overlaid image of fluorescent and bright-field image is shown in the left panel, and the fluorescent (blue, H_2_S) and bright-field image are shown in the middle and right panel, respectively. Bars: 50 μm.

**
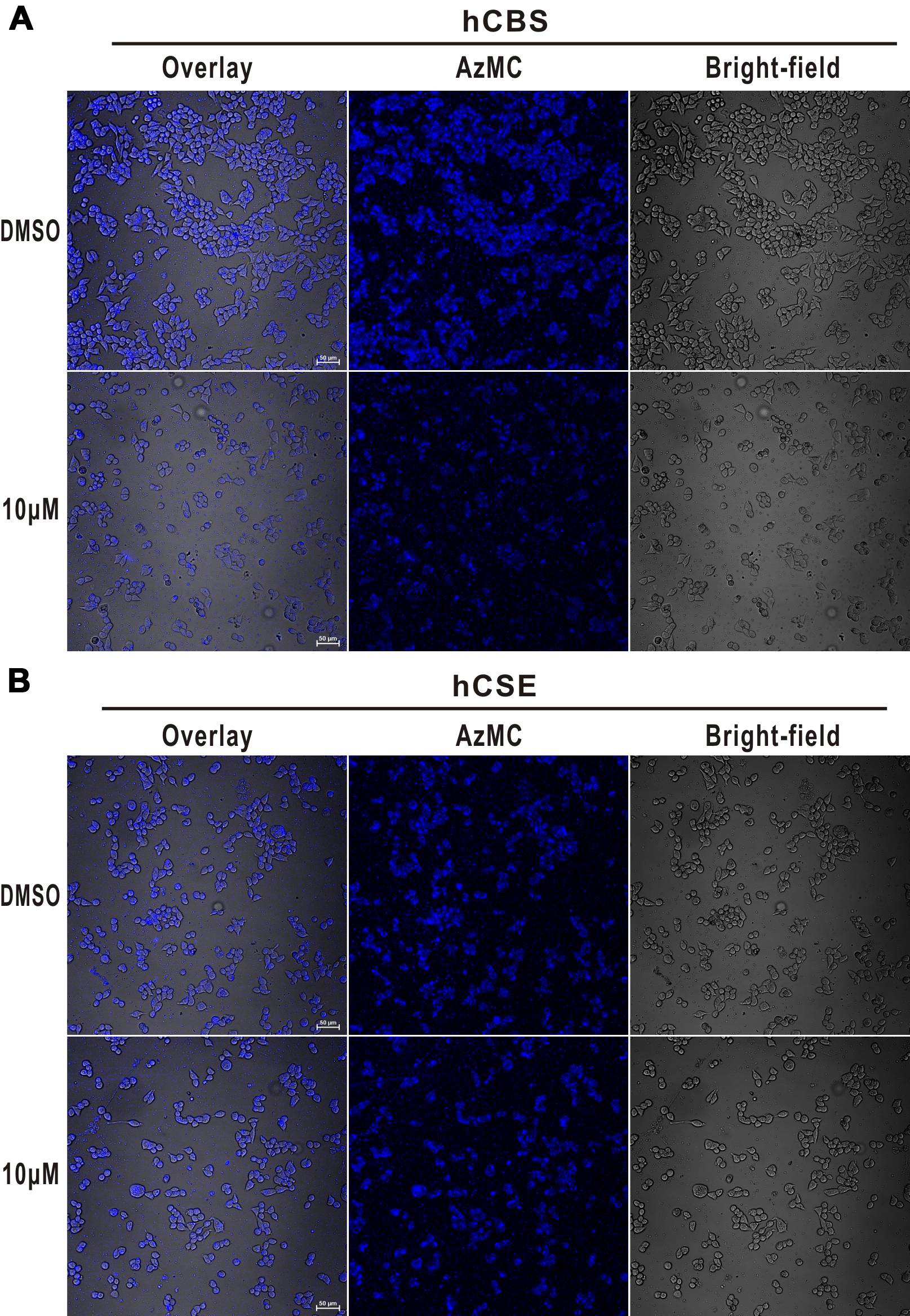
**

Figure S4. Visualization of H_2_S in HEK293T cells expressing of hCBS or hCSE by AzMC probe under confocal microscope**.** CH004 significantly decreased the production of H_2_S in HEK293T cells overexpressing hCBS but not in the cells overexpressing hCSE. HEK293T cells were transiently transfected with hCBS (**A**) or hCSE plasmids (**B**) for 24 h, and followed by the incubation with DMSO or 10 μM CH004 for additional 8 h. After staining with 50 μM H_2_S-specific probe AzMC (Materials and Methods), the fluorescent images of H_2_S (blue) were taken using a Nikon-A1Si confocal microscope. The overlaid images of fluorescent and bright-field images are shown in the left panel, and the corresponding fluorescent (blue, H_2_S) and bright-field images are shown in the middle and right panels, respectively. Bars: 50 μm.


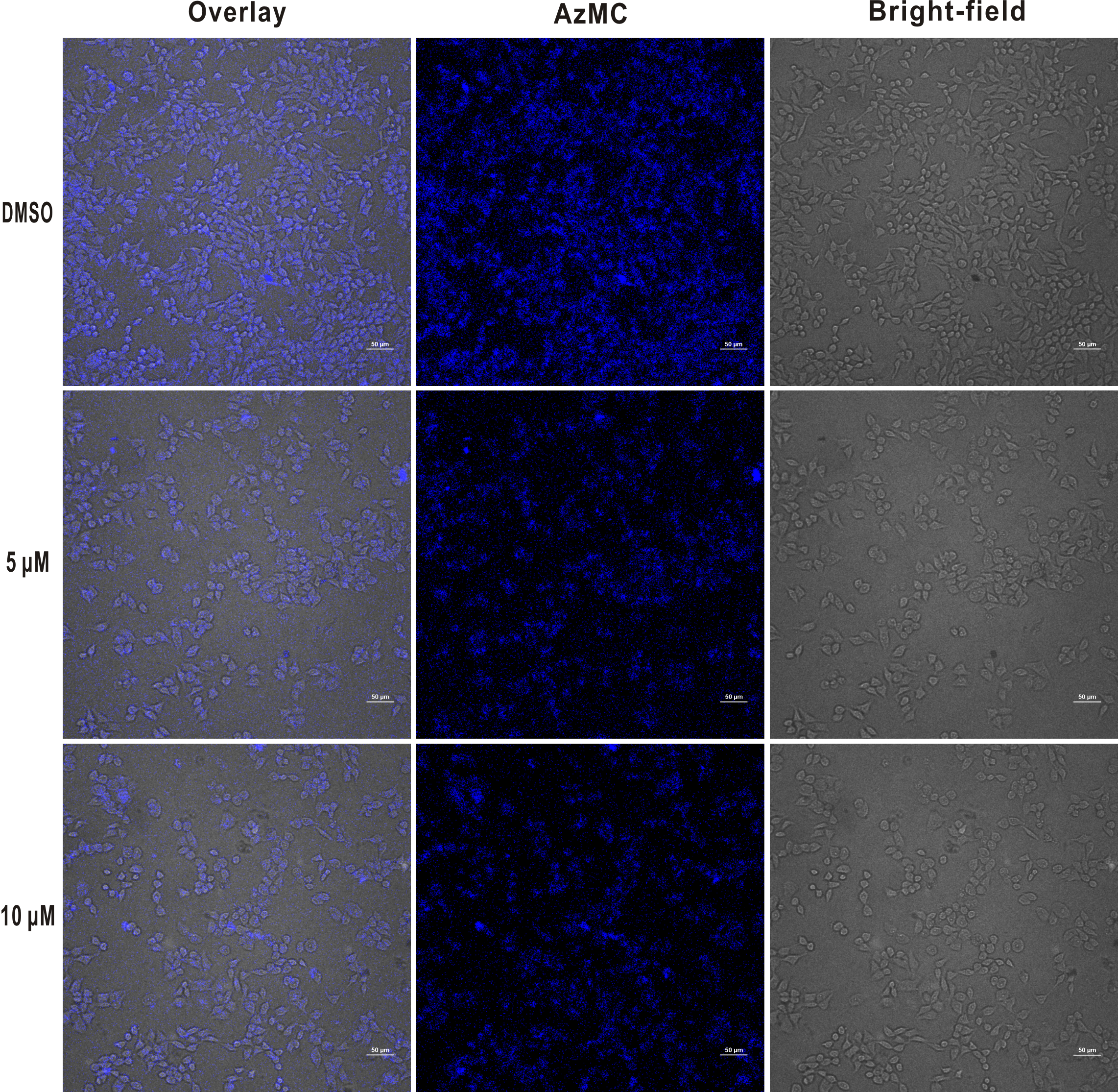


Figure S5. CH004 dose-dependently decreased the H_2_S level in HEK293T cells overexpressing hCBS WT**.** HEK293T cells stably expressing hCBS WT (293-CBS WT) were treated with 0 (DMSO control), 5 or 10 μM CH004 for 8 h before staining with the AzMC to detect H_2_S. The overlaid images of fluorescent and bright-field images are shown in the left panel, and the corresponding fluorescent (blue, H_2_S) and bright-field images are shown in the middle and right panels, respectively. Bars: 50 μm.


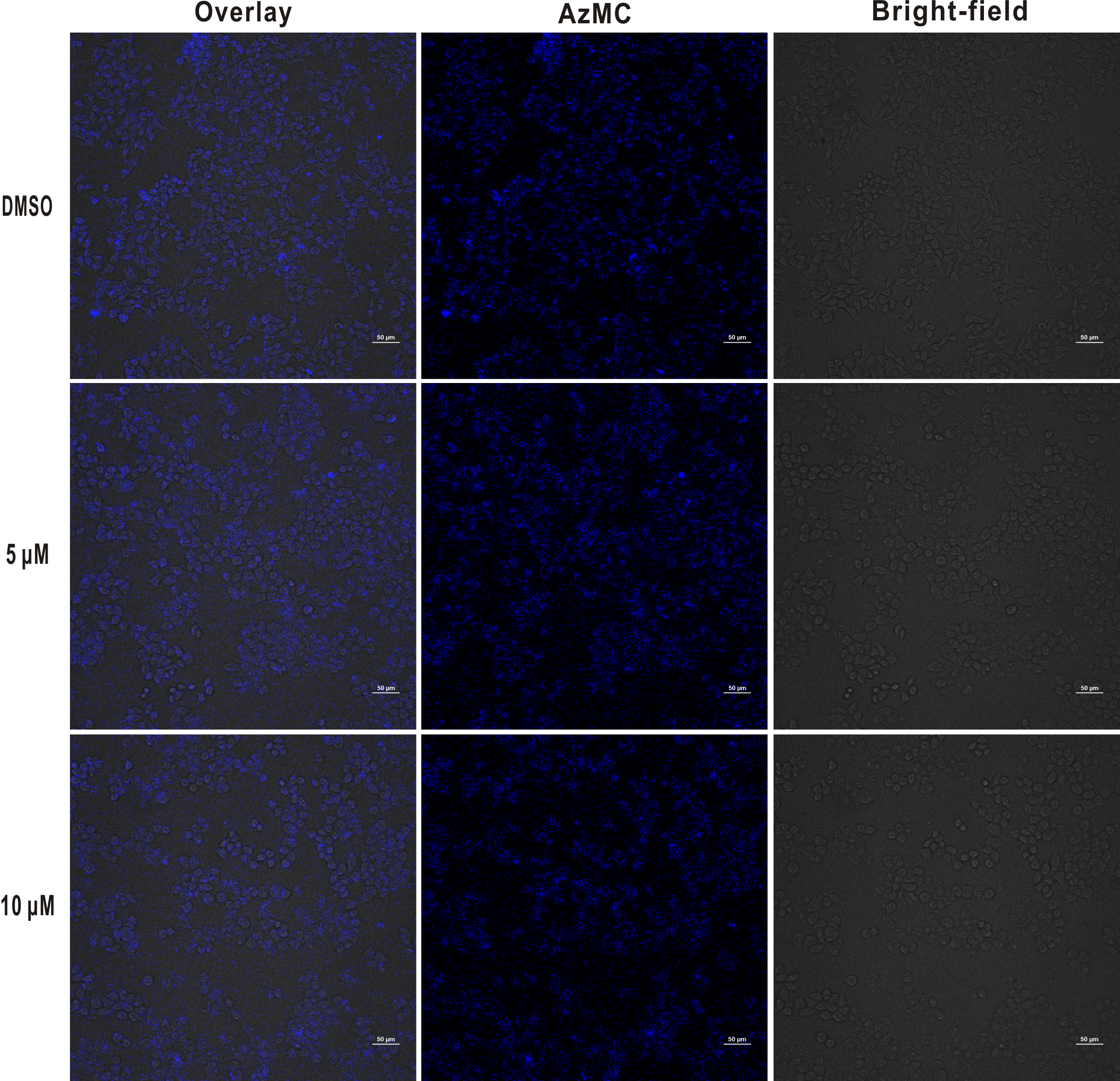


Figure S6. CH004 hardly affected the H_2_S level in HEK293T cells expressing hCBS Q222A mutant. HEK293T cells stably expressing CBS Q222A mutant (293T-CBS Q222A) were treated with 0 (DMSO control), 5 or 10 μM CH004 for 8 h before staining with the AzMC to detect H_2_S. The overlaid images of fluorescent and bright-field images are shown in the left panel, and the corresponding fluorescent (blue, H_2_S) and bright-field images are shown in the middle and right panels, respectively. Bars: 50 μm.


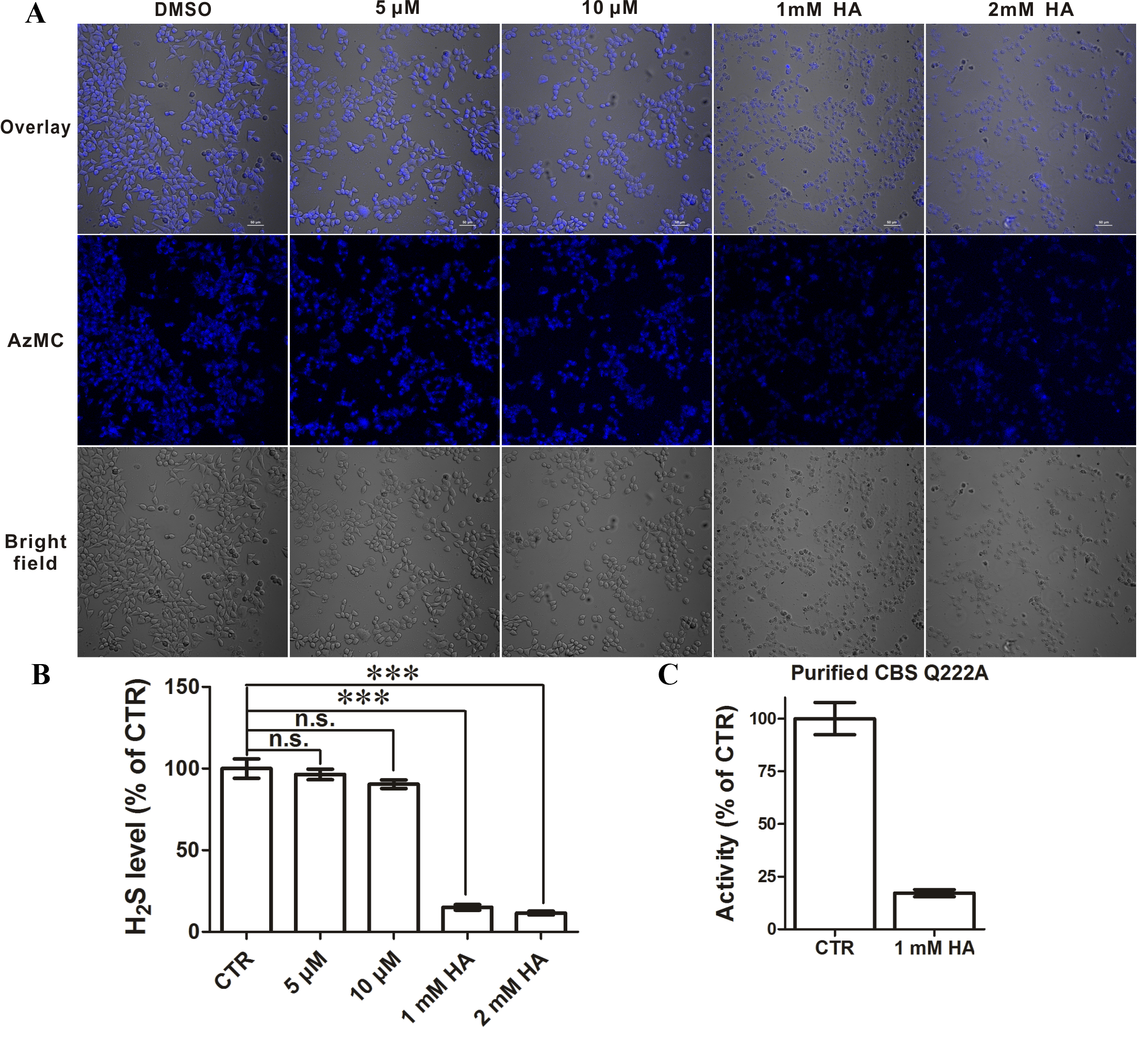
Figure S7. The CH004-insensitive CBS Q222A mutant can be inhibited by HA. (**A**) HEK293T cells stably expressing CBS Q222A mutant (293T-CBS Q222A) were treated with 0 (DMSO, control), 5 or 10 μM of CH004, 1 mM or 2 mM of HA for 8 h before staining with the AzMC to detect H_2_S. The overlaid images of fluorescent and bright-field images are shown in the upper panel, and the corresponding fluorescent (blue, H_2_S) and bright-field images are shown in the middle and lower panels, respectively. Bars: 50 μm. (**B**) Quantification of the mean fluorescent density of H_2_S (blue). The intensity of blue light of the fluorescent images was quantified by ImageJ software, quantified and deduced the background signal in HEK293T-EV cells (Supplementary Figure S3B), normalized by the corresponding cell area and expressed as percentages of control (DMSO group, 100%). Means ± SDs (n=3). One-way ANOVA with Bonferroni post-tests was used for the analysis. *, p< 0.05; **, p< 0.01; n.s. no significance. The images are one representative from three independent experiments. (**C**) The inhibitory effect of 1 mM HA on purified CBS Q222A mutant. The assay was performed using 60 mM Cys and 1.2 μM CBS-413 Q222A mutant. The data were expressed as percentages of control (DMSO, 100%). Means ± SDs (n=4).


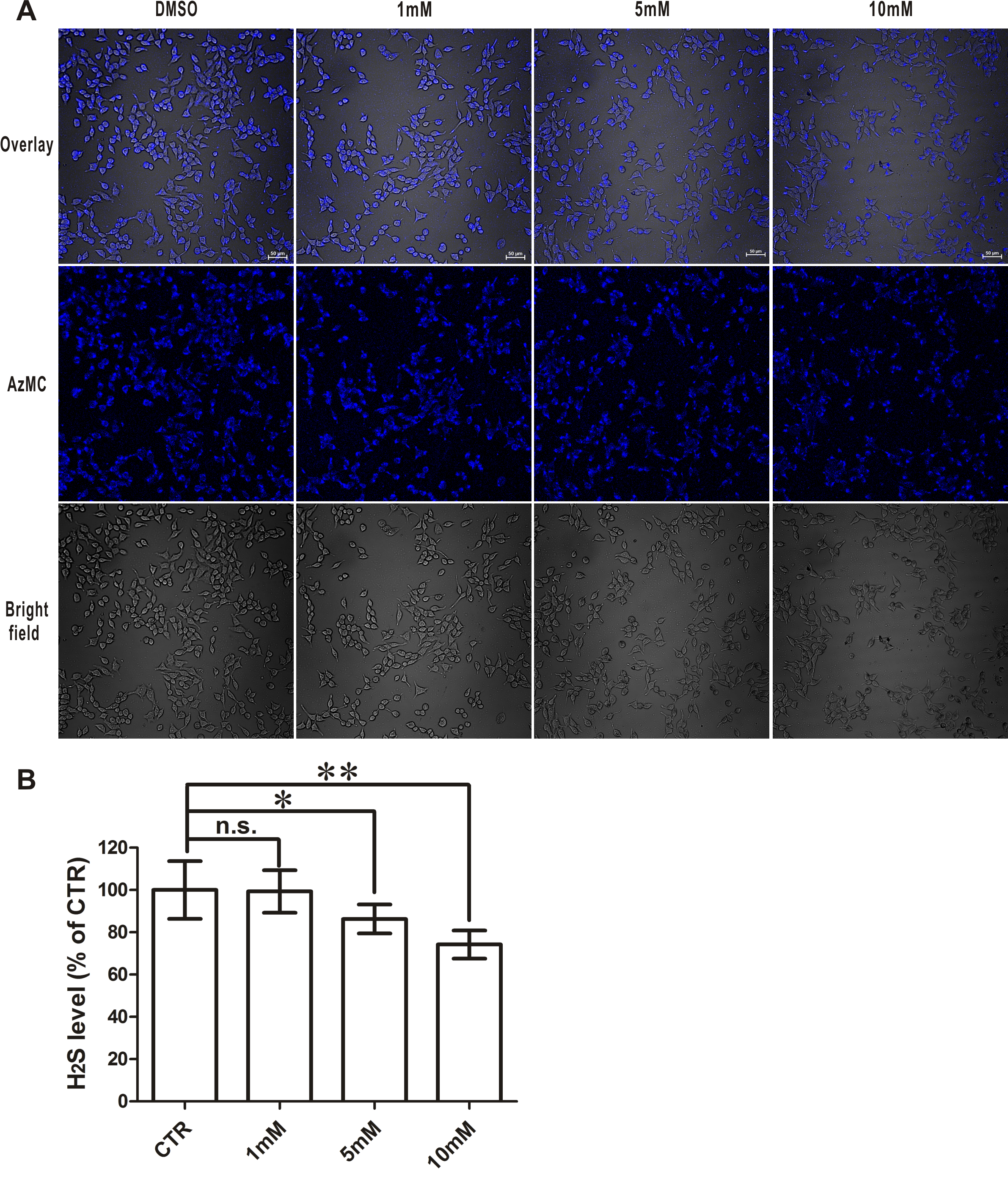


Figure S8. The effect of AOAA on the level of H_2_S in HEK293T cells expressing hCBS WT**.** (**A**) AOAA weakly inhibits the H_2_S production in HEK293T cells expressing hCBS WT. HEK293T cells stably expressing of hCBS (293T-CBS WT cells) were treated with 0 (DMSO control), 1, 5 or 10 mM AOAA for 8 h before staining with 50 μM AzMC to detect H_2_S (Materials and Methods). The overlaid images of fluorescent and bright-field images are shown in the upper panel, and the corresponding fluorescent (blue, H_2_S) and bright-field images are shown in the middle and lower panels, respectively. Bars: 50 μm. (**B**) Quantification of the mean fluorescent density of H_2_S (blue). The intensity of blue light of the fluorescent images was accordingly quantified by ImageJ software (see above), normalized by the corresponding cell area, compared and expressed as percentages of control (DMSO group, 100%). Means ± SDs (n=3). One-way ANOVA with Bonferroni post-tests was used for the analysis. *, p< 0.05; **, p< 0.01; n.s. no significance. The images are one representative from three independent experiments.


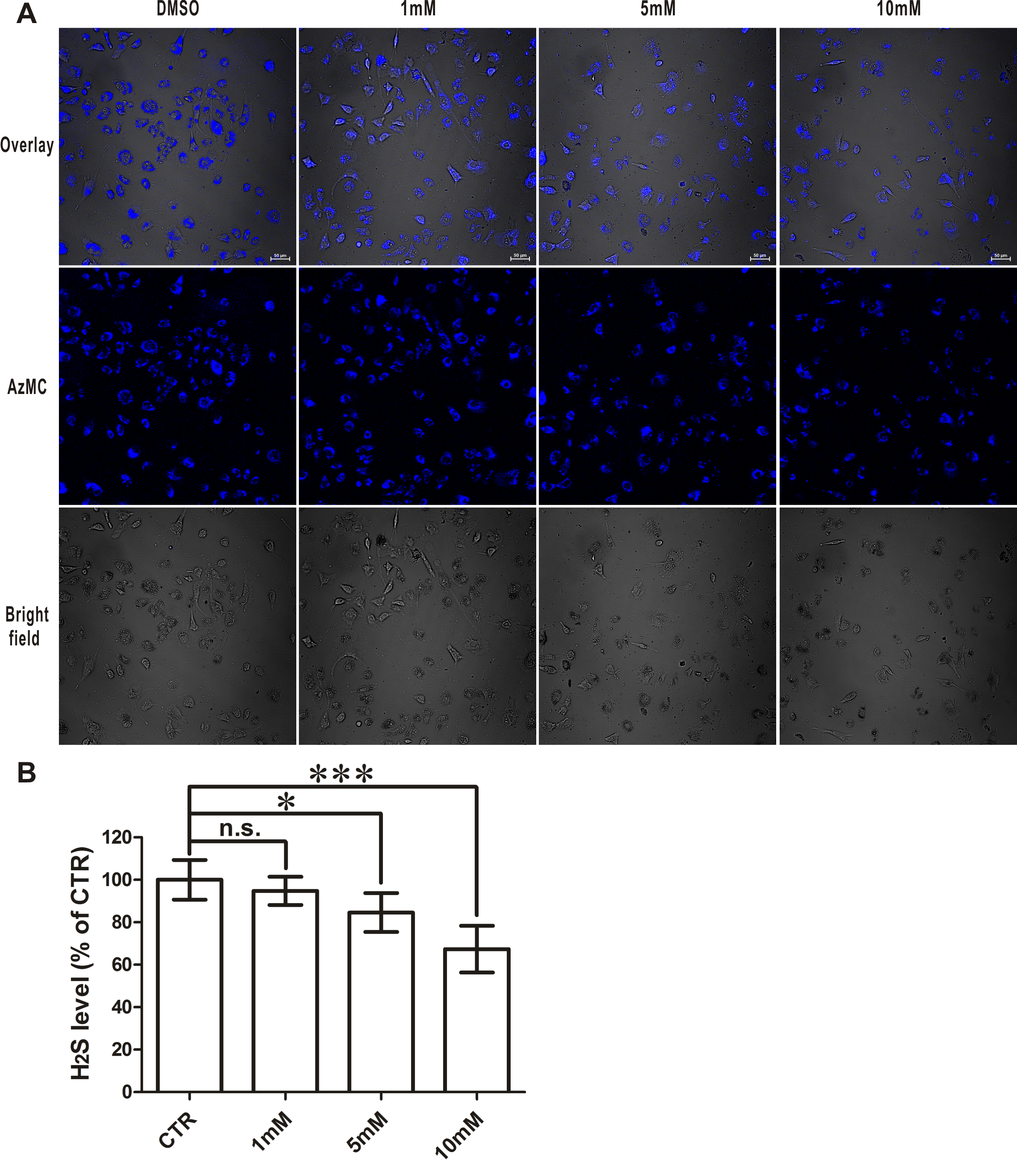


Figure S9. The effect of AOAA on the level of endogenous H_2_S in HepG2 cells**.** (**A**) AOAA impotently decreases the production of endogenous H_2_S in HepG2 cells. HepG2 cells were incubated with 0 (DMSO control), 1, 5 or 10 mM AOAA for 8 h before staining with 50 μM AzMC. Upper panels, the overlaid images; middle panels, the corresponding fluorescent images (blue, H_2_S); the lower panels, the bright-field images. Bars: 50 μm. (**B**) Quantification of the mean fluorescent density of H_2_S (blue). The intensity of H_2_S was accordingly quantified and normalized by correcting for the background signal outside cells. Data was expressed as percentages of the control (DMSO group, 100%). Means ± SDs (n=3). One-way ANOVA with Bonferroni post-tests was used for the analysis. *, p< 0.05; ***, p< 0.001; n.s. no significance. The images are one representative from three independent experiments.


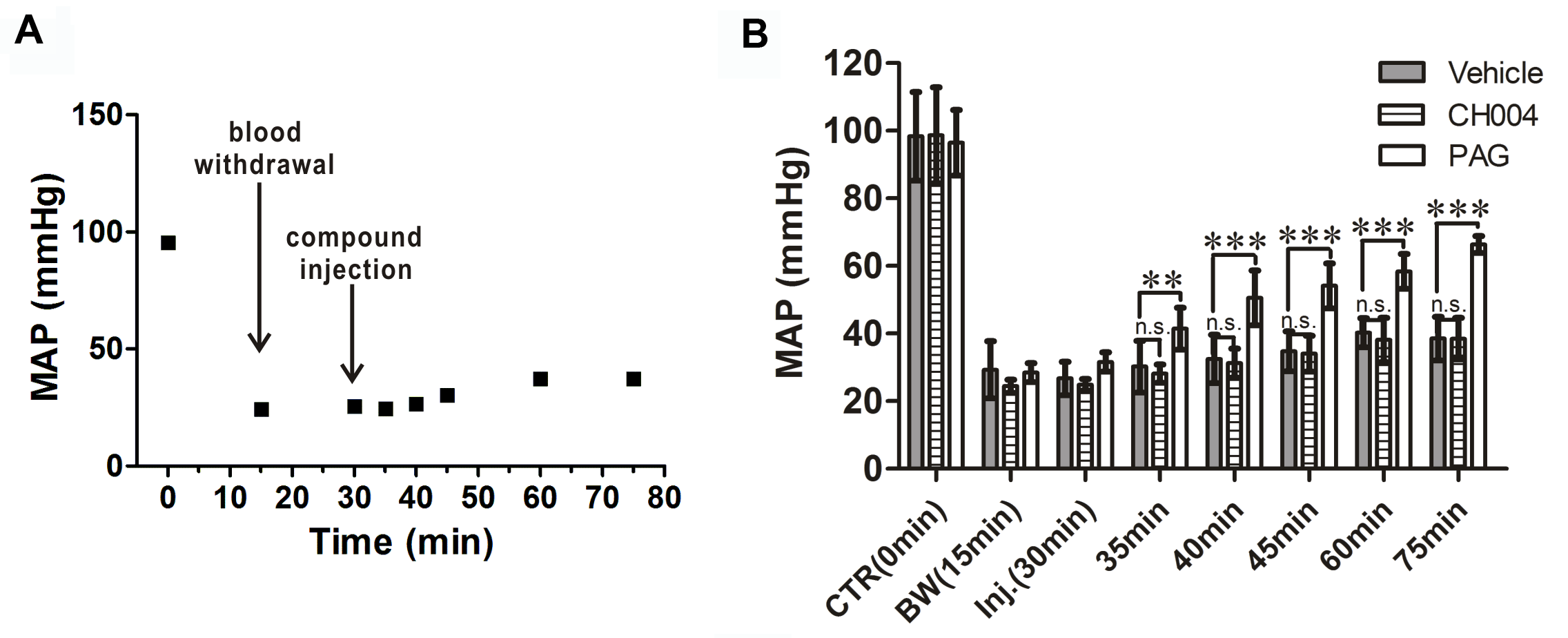


**Figure S10. CH004 didn’t restore the blood pressure in the rat hemorrhagic shock model.** (**A**) The scheme for the hemorrhagic shock experiment in rats. Mean Arterial Blood Pressure, MAP. (**B**) CH004 does not affect the blood pressure in rats. The hemorrhagic shock model was constructed by blood withdrawing (BW) from the carotid artery of anesthetized male Sprague-Dawley rats (280-320 g), and the mean arterial blood pressure (MAP) was monitored by an MP30B-CE pressure transducer (BIOPAC, Santa Barbara, CA, USA). After a stabilizing period of 15 min, the rats were cannulated to withdraw blood (7-9 ml) until the MAP decreased to ~ 25 mmHg. Then, 15 min later, CH004 (3 mg/kg), PAG (50 mg/kg) or vehicle (2% DMSO in saline) was intraperitoneally administered to the rats, and the MAP was monitored continuously for another 45 min. MAP was continually recorded during the experiments. Means ± SDs (n=6). Statistical analyses were performed on the raw data for each group by two-way ANOVA with Bonferroni post-tests. **, p < 0.01; ***, p < 0.001. n.s. no significance.


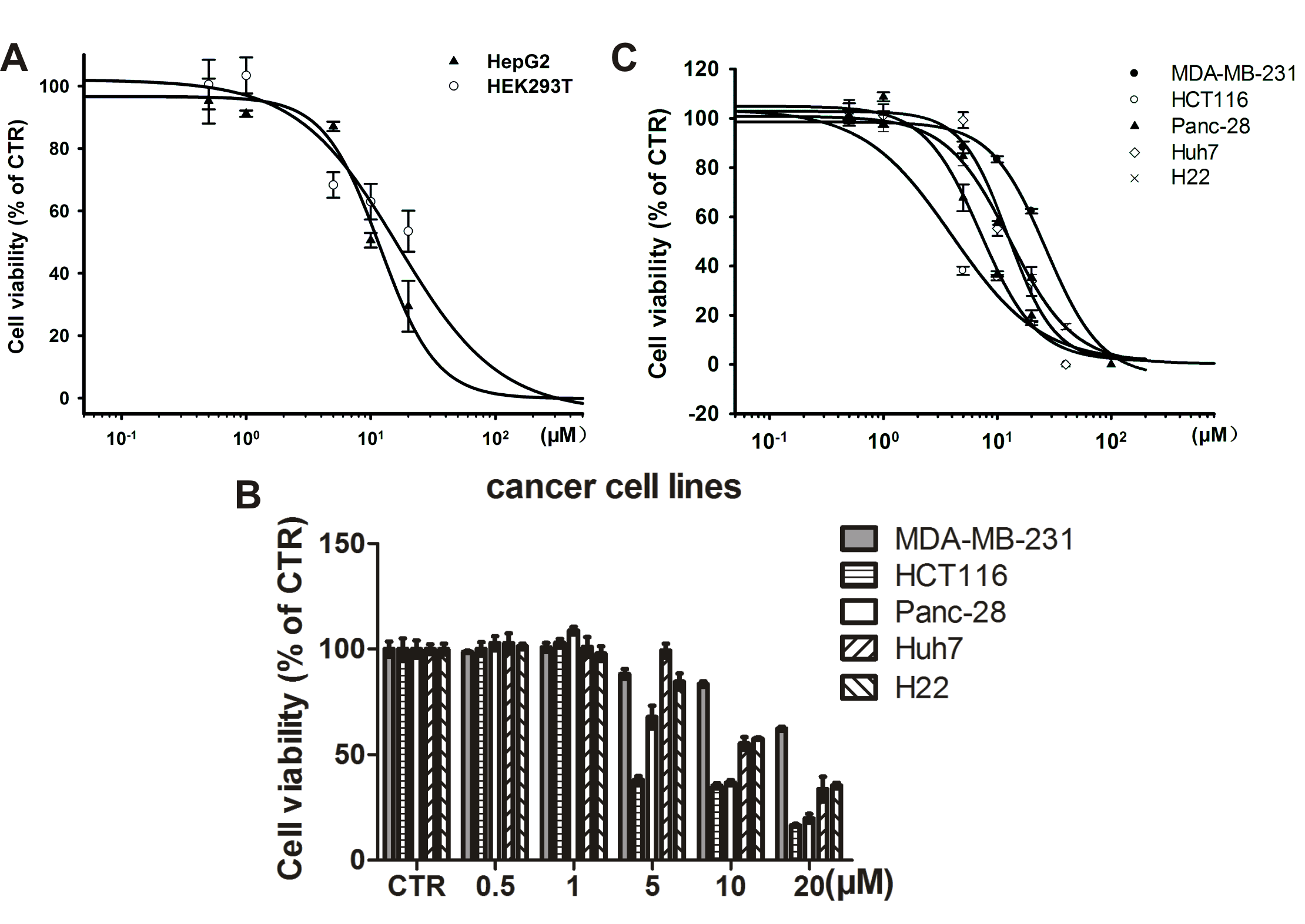


Figure S11. The effects of CH004 on cell viability in various types of cancer cell lines**.** (**A**) Dose-dependent curves of CH004 on cell viability of HepG2 cells (▲), HEK293T cells (○). Percentages of cell viability of CH004 obtained in Figure 4a were fitted to the non-linear equation using GraphPad Prism 5 to get the IC_50_ values of CH004 in respective cell lines. Means ± SDs (n=3). IC_50_ values were 11.3 ± 1.3 μM for HepG2 cells, 16.6 ± 5.3 μM for HEK293T. (**B**) MDA-MB-231 (breast cancer cell cline), HCT116 (colon cancer), Panc-28 cells (pancreas cancer), Huh7 cells (hepatocellular carcinoma) and H22 (mouse liver cancer) were incubated with DMSO (control) or indicated concentrations of CH004 for 24 h in a 96-well plate before assessing cell viability using the CellTiter96® Aqueous One Solution Cell Proliferation Assay (Promega). The data are shown as percentages of control (DMSO, 100%). Means ± SDs (n=3). (**C**) The dose-dependent curve of CH004 on cell viability. Percentages of cell viability obtained in **B** were fitted to the non-linear equation using GraphPad Prism 5 to get the IC_50_ values of CH004 in respective cell lines. The determined IC_50_ values were 4.1 ± 1.2 μM for HCT116 cells (○), 7.2 ± 0.5 μM for Panc-28 cells (▲), 12.4 ± 1.6 μM for Huh7 cells (◊), 25.8 ± 3.2 μM for MDA-MB-231 cells (●) and 12.9 ± 0.7 μM for H22 cells (×). The experiments were independently repeated twice in triplicates and a representative experiment is shown.


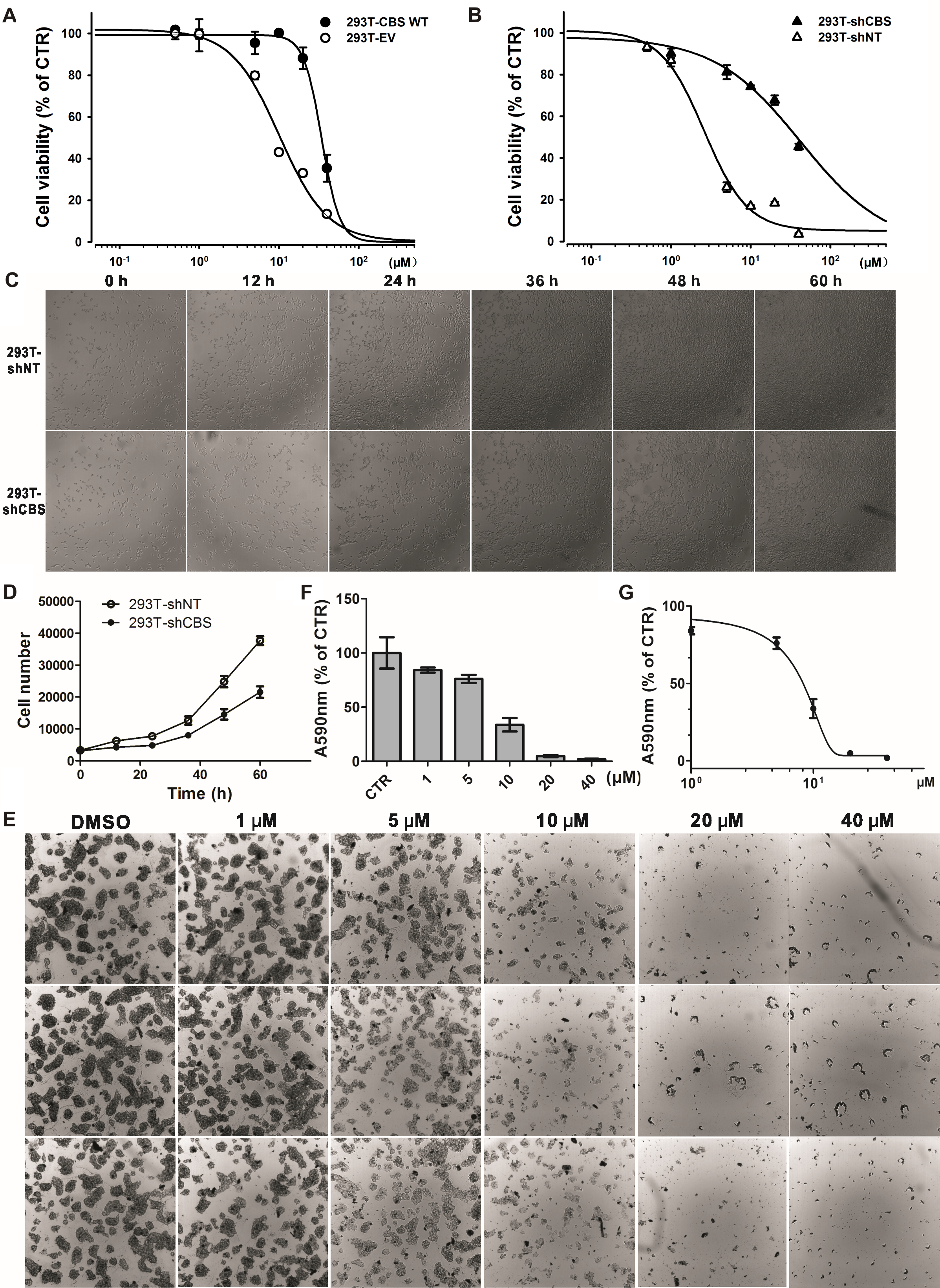


Figure S12. CH004 inhibits the proliferation of cells via specifically targeting hCBS**.** (**A**-**B**) Dose-dependent curves of CH004 on the cell viability of CBS-stably-expressed HEK293T (**A**) or CBS-knockdown cells (**B**). Percentages of cell viability of CH004 obtained in Figure 4b were fitted to the non-linear equation using GraphPad Prism 5 to get the IC_50_ values of CH004 in respective cell lines (●, 293T-CBS WT cells; ○, 293T-EV; ▲, 293T-shCBS; △, 293T-shNT). Means ± SDs (n=3). IC_50_ values were 35.5 ± 1.2 μM and 9.6 ± 1.6 μM for 293T-CBS WT and 293T-EV cells (**A**), and 43.2 ± 8.3 μM and 2.6 ± 0.5 μM for 293T-shCBS and 293T-shNT (**B**), respectively. (**C-D**) ShRNA knock-down of CBS significantly reduces the proliferation rate of HEK293T cells. HEK293T cells stably expressing of the lentiviral shRNA targeting CBS (shCBS) or control shRNA (shNT) were seeded at the density of ~ 3,000 cells per well in 96-well plates and the cell images in the bright field were acquired at indicated times by Image Xpress Micro® XLS (Molecular Devices, Sunnyvale, CA) under 10 × objective lens. Representative images of two independent experiments (n = 6 for each experiment) are shown in **C**. The cell numbers of images under 4 × objective lens of one representative experiment were calculated by Image Xpress Software and the data is shown in **D**. Means ± SDs (n=6). (**E-G**) CH004 dose-dependently suppressed the colony formation of HepG2 cells. HepG2 cells were seeded in a 96-well plate at a density of 20000 cells per well and cultured for 24 h before treatment with indicated concentrations of CH004 for another 24 h. The colonies were stained with crystal violet and quantified by measuring the absorbance at 590 nm. The images for the colonies were taken by Image Xpress Micro® XLS (Molecular Devices) using 10 × objective lens and shown in **E**. The data of CH004 at each concentration are expressed as percentages of control (DMSO, 100%) and shown in **F**. Dose-dependent curve of CH004 (**G**) was generated by fitting the data (**F**) to the non-linear equation using GraphPad Prism 5 to get the IC_50_ (~ 7.9 ± 2.3 μM). Means ± SDs (n=3). The colony formation experiment was independently repeated twice in triplicates.


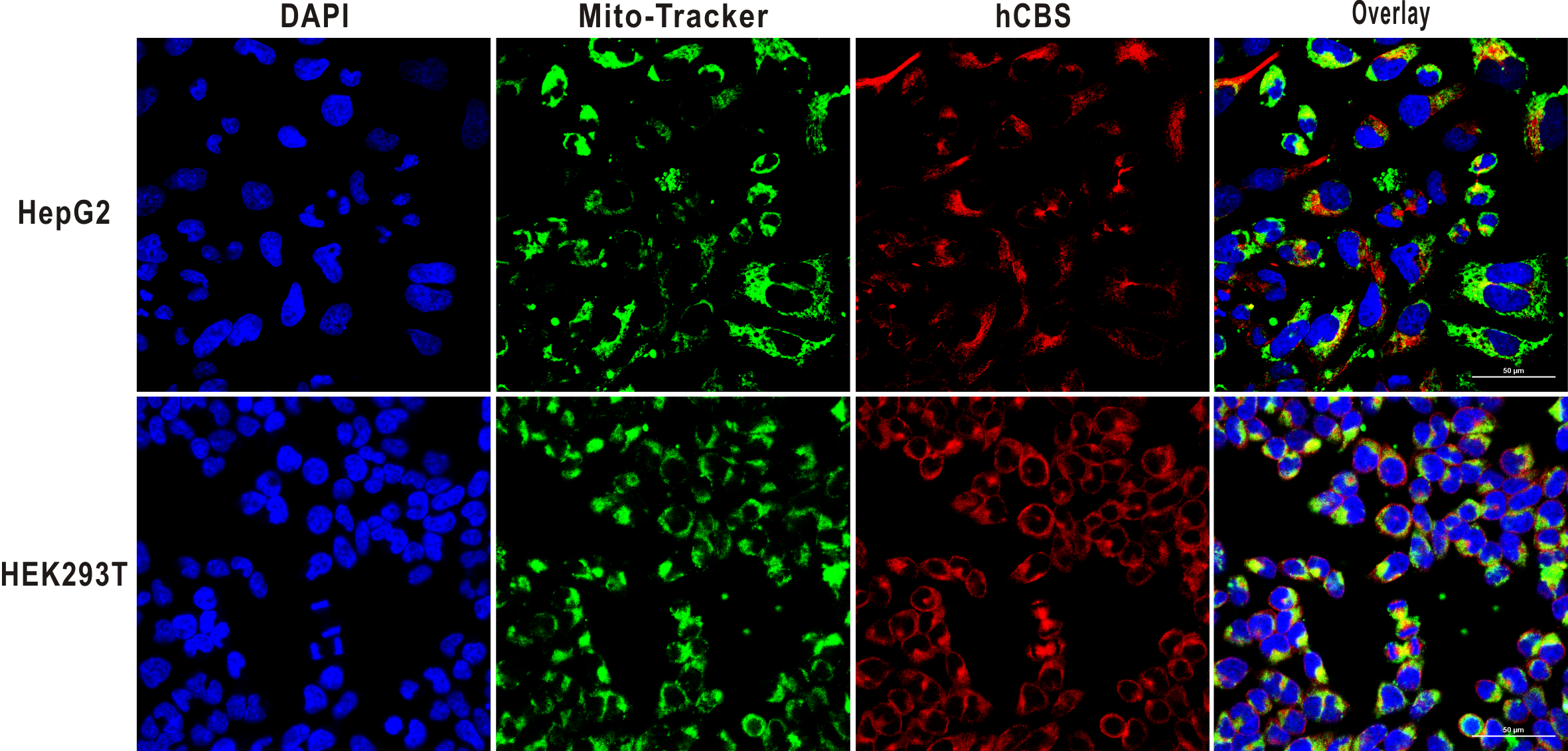


Figure S13. hCBS co-localizes with mitochondria in HepG2 and HEK293T cells. HepG2 and HEK293T cells were stained with an anti-CBS antibody (A-2, 1:200; Santa Cruz) for hCBS (red), Mito-tracker® Green for mitochondria (green) or DAPI for the nucleus (blue). The immunofluorescence images were taken using a Nikon-A1Si confocal microscope. Yellow, colocalization of CBS (red) and mitochondria tracker (green). Bars: 50 μm. All images are representative of three independent experiments.


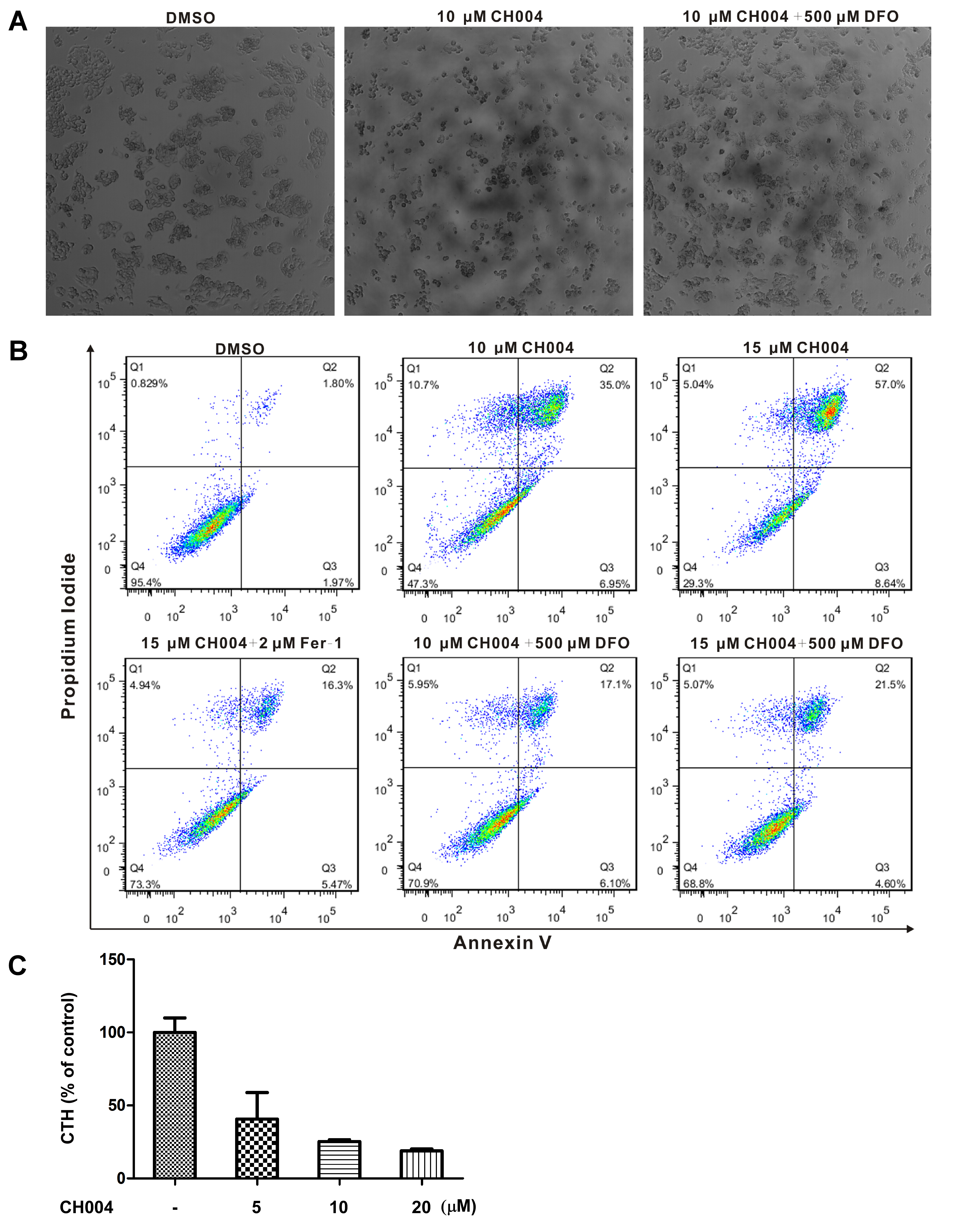


Figure S14. CH004-induced cell death was prevented by ferroptosis inhibitors and CH004 reduces the intracellular amount of **cystathionine** in HepG2 cells. HepG2 cells were treated under the conditions described in Figure 6d. After treatment with inhibitors for 24 h, HepG2 cells were taken images by Image Xpress Micro® XLS using 10 × objective lens (**A**) or analyzed using a FITC Annexin V Apoptosis Detection Kit on an LSR Fortessa flow cytometer (BD bioscience; **B**). All images are representative of three independent experiments. (**C**) CH004 dose-dependently inhibits the amount of cystathionine in HepG2 cells. HepG2 cells were treated with indicated DMSO or CH004 for 8 h in the presence of 100 μM Hcys and 100 μM Ser substrates, the cells were then lysed and the supernatant was collected for the quantitative analysis of CTH with LC-MS/MS (Materials and Methods). The amount of CTH was normalized with the corresponding protein amount (BCA, Pierce) and displayed as a percentage of control (DMSO, 100%). Means ± SDs (n=2). The experiment was independently repeated twice and one representative result is present.


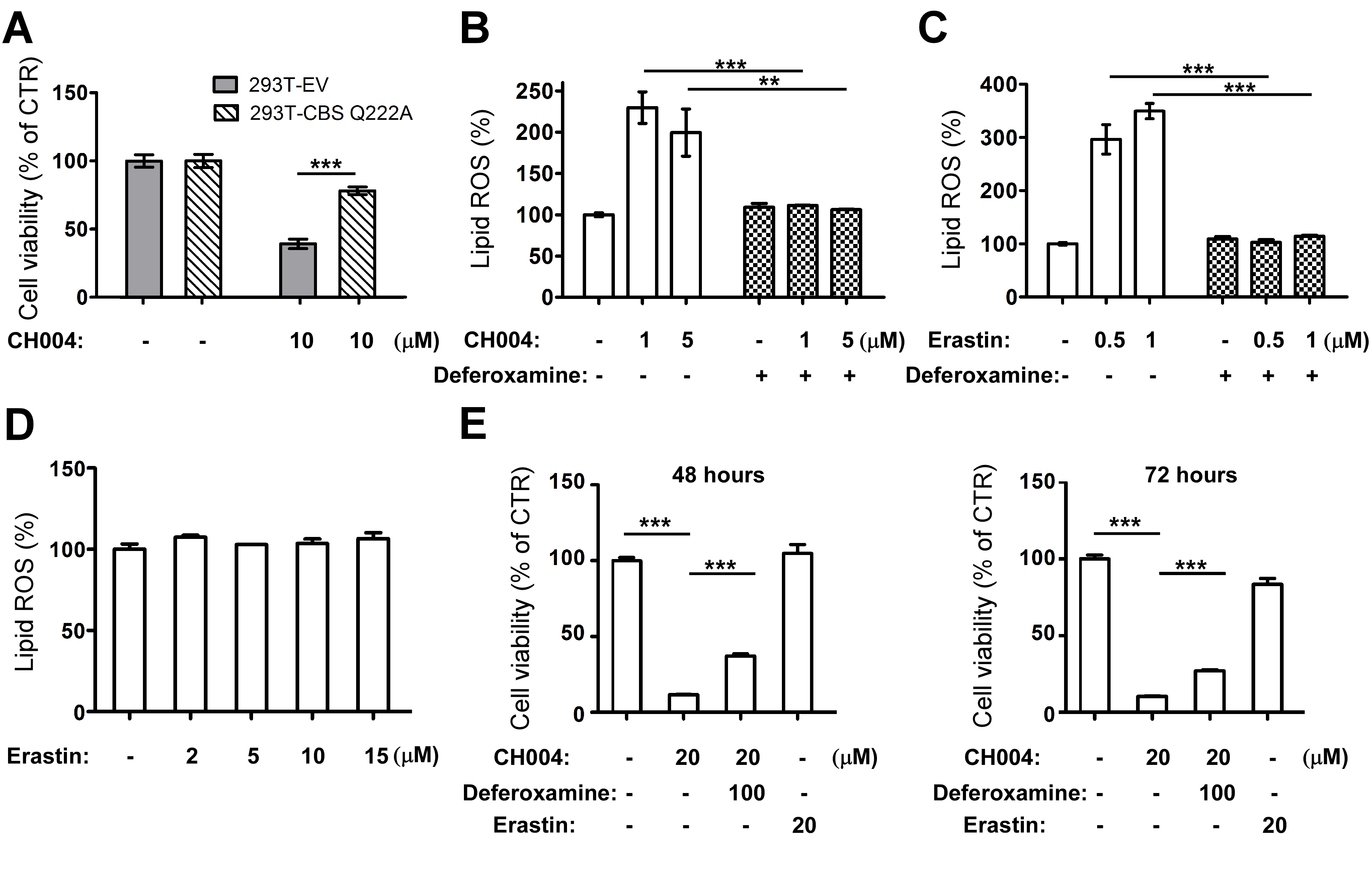


Figure S15. The mode of actions for CH004 in cells. (**A**) Expression of CH004-insensitive CBS Q222A mutant partially rescues the cell death triggered by CH004 in HEK293T cells. Indicated HEK293T stable cell lines were treated with CH004 at a concentration of 10 μM for 24 h, and the cell viability was then measured using the CellTiter96® Aqueous One Solution Cell Proliferation Assay (Promega). Means ± SDs (n=3). (**B-C**) CH004 or erastin triggers ferroptosis in mouse embryonic fibroblasts (MEF). WT MEF cells were treated with CH004 or erastin at indicated concentrations in the presence or the absence of 500 μM DFO for 24 h before measuring the lipid ROS with 2 μM BODIPY® 581/591 C11 (Materials and methods). (**D**) Erastin does not induce ferroptosis in HepG2 cells. Erastin at micromolar concentrations were incubated with HepG2 cells for 24 h before measuring the lipid ROS (see above and Materials and methods). The FACS data of lipid ROS (green fluorescent) was displayed as percentages of control (DMSO). Means ± SDs (n=3). (**E**) DFO partially rescues the cell death caused by a high dose of CH004 at long incubation times. CH004 or erastin at a concentration of 20 μM was incubated with HepG2 cells for 48 h or 72 h in the presence or the absence of 100 μM DFO. The cell viability was accordingly quantified using the CellTiter96® Aqueous One Solution Cell Proliferation Assay (Promega) and the results were shown as percentages of DMSO (control, 100%). Means ± SDs (n=3). All the experiments were independently repeated at least twice and one representative result is present.

**Table S1 The inhibitory effects of CH004 on purified hCBS, hCSE and hDDC.**

| Name | Chemical structure | IC_50_ (μM) | | |
| --- | --- | --- | --- | --- |
|  |  | hCBS-413 | hCSE (Fold selectivity^1^) | hDDC (Fold selectivity^1^) |
| CH004 |  | 1.0±0.2  0.59±0.1^2^  1.7±0.3^3^ | 29.9±5.6  (29.9) | > 400  (> 400) |

^1^The fold-change in selectivity between the IC_50_ of hCSE or hDDC and of hCBS-413.

^2^The IC_50_ were determined for hCBS-FL.

^3^The IC_50_ were determined for hCBS-FL by Methyl Blue method (ref. 1).

**Table S2. Inhibitory effects of CH004 on hCBS WT and mutants.** The IC_50_ values of CH004 for CBS was determined according to the following conditions, i.e. 150 nM purified CBS for WT, S147A, Q222A, Y223F mutants or 2 μM CBS for T146A mutant, in the presence of 4 mM Hcys and 4 mM Cys as well as 50 mM Tris-HCl (pH 8.6) ^2^.

| hCBS | IC_50_ (μM) | IC_50_ fold to WT |
| --- | --- | --- |
|
| WT | 1.2±0.05 | 1 |
| T146A | 5±1.5 | 4.2 |
| S147A | 5.9±0.8 | 4.9 |
| Q222A | 27.6±0.9 | 23.0 |
| Y223F | 4.2±1.0 | 3.5 |

Table S3. Primer sequence**s**

| **No.** | **Primer** | **Usage** |
| --- | --- | --- |
| 1 | 5'-CACGATTATCGAGCCGGCATCCGGGAACACCGGGATCGGGC-3' | 5' primer for constructing T146A mutant |
| 2 | 5'-GCCCGATCCCGGTGTTCCCGGATGCCGGCTCGATAATCGTG-3' | 3' primer for constructing T146A mutant |
| 3 | 5'-CACGATTATCGAGCCGACAGCCGGGAACACCGGGATCGGGC-3' | 5' primer for constructing S147A mutant |
| 4 | 5'-GCCCGATCCCGGTGTTCCCGGCTGTCGGCTCGATAATCGTG-3' | 3' primer for constructing S147A mutant |
| 5 | 5'-CCCCAATTCTCACATCCTAGACGCGTACCGCAACGCCAGCAACCCCC-3' | 5' primer for constructing Q222A mutant |
| 6 | 5'-GGGGGTTGCTGGCGTTGCGGTACGCGTCTAGGATGTGAGAATTGGGG-3' | 3' primer for constructing Q222A mutant |
| 7 | 5'- CCCCAATTCTCACATCCTAGACCAGTTCCGCAACGCCAGCAACCCCC-3' | 5' primer for constructing Y223F mutant |
| 8 | 5'-GGGGGTTGCTGGCGTTGCGGAACTGGTCTAGGATGTGAGAATTGGGG-3' | 3' primer for constructing Y223F mutant |

REFERENCES

1. Stipanuk, M. H. & Beck, P. W. Characterization of the enzymic capacity for cysteine desulphhydration in liver and kidney of the rat. *Biochem J* **206**, 267-277 (1982).

2. Zhou, Y. et al. High-throughput tandem-microwell assay identifies inhibitors of the hydrogen sulfide signaling pathway. *Chem Commun (Camb)* **49**, 11782-11784 (2013).
